# Proteins Related to the Type I Secretion System Are Associated with Secondary SecA_DEAD Domain Proteins in Some Species of *Planctomycetes*, *Verrucomicrobia*, *Proteobacteria*, *Nitrospirae* and *Chlorobi*

**DOI:** 10.1371/journal.pone.0129066

**Published:** 2015-06-01

**Authors:** Olga K. Kamneva, Saroj Poudel, Naomi L. Ward

**Affiliations:** 1 Department of Biology, Stanford University, Stanford, CA, 94305–5020, United States of America; 2 Computer Science Department, Montana State University, Bozeman, MT, 59717, United States of America; 3 Department of Molecular Biology, University of Wyoming, Laramie, WY, 82071, United States of America; 4 Department of Botany, University of Wyoming, Laramie, WY, 82071, United States of America; 5 Program in Ecology, University of Wyoming, Laramie, WY, 82071, United States of America; The Scripps Research Institute, UNITED STATES

## Abstract

A number of bacteria belonging to the PVC (*Planctomycetes*-*Verrucomicrobia*-*Chlamydiae*) super-phylum contain unusual ribosome-bearing intracellular membranes. The evolutionary origins and functions of these membranes are unknown. Some proteins putatively associated with the presence of intracellular membranes in PVC bacteria contain signal peptides. Signal peptides mark proteins for translocation across the cytoplasmic membrane in prokaryotes, and the membrane of the endoplasmic reticulum in eukaryotes, by highly conserved Sec machinery. This suggests that proteins might be targeted to intracellular membranes in PVC bacteria via the Sec pathway. Here, we show that canonical signal peptides are significantly over-represented in proteins preferentially present in PVC bacteria possessing intracellular membranes, indicating involvement of Sec translocase in their cellular targeting. We also characterized Sec proteins using comparative genomics approaches, focusing on the PVC super-phylum. While we were unable to detect unique changes in Sec proteins conserved among membrane-bearing PVC species, we identified (1) SecA ATPase domain re-arrangements in some *Planctomycetes*, and (2) secondary SecA_DEAD domain proteins in the genomes of some *Planctomycetes*, *Verrucomicrobia*, *Proteobacteria*, *Nitrospirae* and *Chlorobi*. This is the first report of potentially duplicated SecA in Gram-negative bacteria. The phylogenetic distribution of secondary SecA_DEAD domain proteins suggests that the presence of these proteins is not related to the occurrence of PVC endomembranes. Further genomic analysis showed that secondary SecA_DEAD domain proteins are located within genomic neighborhoods that also encode three proteins possessing domains specific for the Type I secretion system.

## Background

The *Planctomycetes*, *Verrucomicrobia*, *Chlamydiae* (PVC) super-phylum is recognized as a group of established (*Planctomycetes*, *Verrucomicrobia*, *Chlamydiae* and *Lentisphaerae*) and candidate (*OP3* and *Poribacteria*) bacterial phyla [1], although a recent analysis identified *Poribacteria* as a separate phylogenetic group [2]. FtsZ-independent cell division in planctomycetes and chlamydiae is one property that distinguishes these groups from typical Gram-negative bacteria [1]. Cell wall composition is also atypical; there are no reports of peptidoglycan in planctomycetes, although it has recently been detected in chlamydial cell walls [3]. In contrast, canonical Gram-negative cell wall and cell division machinery is found in contemporary *Verrucomicrobia* and *Lentisphaerae*, and genomic analysis suggests that it was present in the PVC ancestor [4], a finding that supports retention of a Gram-negative classification for the super-phylum. Additionally, the PVC super-phylum contains bacteria with unusual cellular organization, featuring intracellular membranes of varying structure [5–7]. These range from a single membrane separating ribosome-containing and ribosome-free parts of the cell in *Pirellula* species, to an extensively invaginated membrane in *Planctomyces* and *Gemmata* species, and numerous membrane vesicles in *Gemmata* [7–9]. From this point forward, we will use the general term “intracellular membranes” to describe this wide variety of structures.

The evolutionary origin of intracellular membranes in PVC bacteria and their relation to the origin of Eukaryotes has fueled extensive discussion in the scientific literature [10, 11]. The membranes have long been proposed to exist in additional to the cytoplasmic membrane, generating a cell plan unique to some PVC organisms [5, 6]. An alternative interpretation posits that the membranes are formed through invagination of the cytoplasmic membrane, providing an extension of canonical cell organization of Gram-negative Bacteria, rather than a unique plan [12].

The biological functions (except for *K*. *stuttgartiensis)* and molecular origins of PVC intracellular membranes are unknown [1]. Electron microscopy studies showed the presence of ribosome-like particles attached to these membranes in some *Planctomycetes* species [13, 7], indicating that in these species the membrane is a site for co-translational protein translocation, canonically mediated by the Sec pathway.

The highly conserved Sec (translocon) pathway mediates co-translational and post-translational translocation of signal peptide-containing proteins across the cytoplasmic membrane in bacteria and archaea, and the membrane of rough endoplasmic reticulum in eukaryotes [14, 15]. The final destinations of proteins transported via Sec in Gram-negative bacteria depend on several additional factors, and include the cytoplasmic membrane, periplasm, outer membrane, cell wall, and extracellular space. The Sec pathway is composed of several proteins. SecYEG constitutes a transmembrane channel, the main component of the translocase [16–18]. The signal recognition particle (SRP) and SRP receptor act together in co-translational translocation and target the ribosome-mRNA-nascent peptide complex to the translocon. SecB is a chaperone which maintains the unfolded state of proteins targeted for post-translational translocation via Sec [19]. Other accessory membrane-bound proteins that increase translocation efficiency are SecD, SecF and YajC [20, 21]. The last component is a SecA ATPase which both participates in post-translational targeting of proteins to the SecYEG channel, and provides transport energy via ATP hydrolysis [22].

Comparative genomic analyses have identified genes potentially associated with PVC cellular organization [23–26]. In a previous paper on genome content evolution in PVC bacteria, we and our colleagues identified a number of genes preferentially present in PVC bacteria possessing intracellular membranes, Kamneva *et al*, 2012 [26]. Within the same study, we performed functional sequence analysis on a sample set of proteins, DUF1501-containing genomic clusters. DUF1501 is a domain of unknown function found in some bacterial genomes but lacking biochemical or any other type of characterization. Several proteins in this sample set were ascertained to possess signal peptides, suggesting that they are targeted to Sec-mediated membrane translocation. However, it is not clear whether the presence of signal peptides, and potential Sec-mediated targeting within cells, is limited to the DUF1501-containing genomic module, or if the same targeting mechanism can be suggested for other proteins associated with PVC intracellular membranes. If the latter is true, then signal peptides should be over-represented among proteins associated with PVC intracellular membranes. Thus the first goal of this study was to conduct a systematic computational analysis of proteins putatively associated with PVC intracellular membranes as reported in Kamneva *et al*, 2012 [26] to assess the distribution of signal peptides within these proteins and potentially predict their targeting mechanism.

Although the Sec pathway is integral to the molecular function of membrane structures across all three domains of life, evolutionary or comparative analysis of this machinery in PVC bacteria has not been carried out. Therefore the second goal of this study was to perform comparative sequence analysis of Sec pathway proteins to detect changes that might be associated with the emergence of PVC intracellular membranes.

## Methods

### Characterization of Sec protein domain composition, and detection of Sec pathway protein homologs

In order to identify protein domains which could be used as markers for various Sec proteins, we analyzed the domain composition of every protein from the Sec-dependent protein export pathway in *Escherichia coli* K-12 MG1655, *Chlamydia muridarum* Nigg, *Bacillus subtilis* subsp. *subtilis* 168, *Nostoc* sp. PCC 7107, *Bifidobacterium bifidum* PRL2010, and *Bacteroides fragilis* NCTC9343, as assigned by homology groups in the KEGG database [27] by searching against the PFAM database [28]. The longest domain present in the protein from all organisms, or a combination of domains, was designated as a protein signature (S1 Table).

A previously analyzed set of genomes from Kamneva *et al*, 2012 [26] was used for initial comparative analysis of Sec proteins. This set contained 315,905 proteins from 99 bacterial genomes, representing 20 bacterial phyla that included all the *Planctomycetes*, *Verrucomicrobia* and *Lentisphaerae* genomes sequenced at the time, as well as representative genomes from phylum *Chlamydiae* and other non-PVC bacterial phyla. This set of genomes is described in detail in the original article Kamneva *et al*, 2012 [26]. Homologs for every Sec pathway component were identified in the 99 bacterial genomes included in the dataset, using signature domains for those components (S1 Table). Protein domain composition was determined for every protein by searching against PFAM, and in the case of SecA proteins, visualized using a custom R script.

### Construction of an extended sequence dataset

In order to identify genomes containing sequences similar to secondary SecA_DEAD domain proteins from PVC genomes, we carried out an initial blast search [29] against GenBank [30] and found some proteobacterial genomes, as well as one member each of the phyla *Nitrospirae* and *Chlorobi*, that encode similar sequences. To provide broader phylogenetic context for our analysis and establish the evolutionary relationship between secondary and canonical SecA ATPases, we assembled a set of distantly related bacterial and archaeal genomes available via GenBank [30]. The leucyl-tRNA synthetase protein sequence was extracted from every genome and used as a marker to filter out closely related genomes. The leucyl-tRNA synthetase gene was selected because it is routinely used as a marker in species tree reconstruction [31, 32] and taxonomic placement of poorly characterized or newly discovered organisms [33]. These sequences were clustered using the blastclust program, applying 80% amino acid identity and 85% alignment extension thresholds. One representative genome from every cluster was included in the extended set of bacterial genomes. We also supplemented this set of divergent bacteria by inclusion of all available *Planctomycetes*, *Lentisphaerae*, and *Verrucomicrobia* genomes, as well as genomes from species within the *Proteobacteria*, *Nitrospirae* and *Chlorobi* determined to possess genes similar to those encoding secondary SecA_DEAD domain proteins in PVC bacteria. The final extended genome dataset contained 2,460,622 proteins from 786 bacteria and archaea. The list of genomes and associated nucleotide accessions is available online: https://www.dropbox.com/sh/koq9oo0bumx4m19/AABavnbOn8zuqawvgAEiG90_a?dl=0

### Protein alignments, phylogeny reconstruction, and domain searches

Additional SecA sequences were extracted from this extended set of genomes using blast search with an E-value threshold of 1e-10, and then further refined using the presence of the signature SecA_DEAD domain, as defined by significant PFAM hits [28].

Extended gene families for genes from the genomic neighborhoods were constructed using blast search against the genome dataset, using an E-value threshold of 1e-10 without additional refinement. Alignments were performed using MUSCLE [34], and the phylogeny for every gene family was reconstructed using FastTree [35] implementing the JTT+I+GAMMA model of evolution [36–38]. Domain composition of every protein was determined by searching against PFAM with the E-value cut-off set to 1, in order to detect even weak domain similarity. Phylogenetic trees and protein domain compositions were visualized using a custom R script. The large-scale SecA phylogeny was visualized using the iTOL web-server [39] showing phylum- or class-level taxonomy for every tip. Alignments, phylogenetic trees and domain annotations are available online: https://www.dropbox.com/sh/koq9oo0bumx4m19/AABavnbOn8zuqawvgAEiG90_a?dl=0

### Signal peptide search and statistical analysis

Signal peptides were predicted for every protein in the dataset using lipoP and signalP programs [40, 41]. LipoP was initially trained on a set of sequences from Gram-negative bacteria [40] but has also been shown to perform well on sequences from Gram-positive bacteria [42]. SignalP was used with the Gram-positive or Gram-negative option depending on the organism; a table listing all the genomes and their cell wall type is available online: https://www.dropbox.com/sh/koq9oo0bumx4m19/AABavnbOn8zuqawvgAEiG90_a?dl=0

Twin-arginine signal peptides were predicted using TAT_signal (PF10518) PFAM domain. Every gene family in the dataset was classified as having a signal peptide or a twin-arginine signal peptide if it was detected in at least 50% of proteins within the family. The association of every gene family with the presence of PVC intracellular membranes was obtained from Kamneva *et al*, 2012 [26]. P-values for the association between the presence of signal peptides and intracellular membranes were obtained using a hypergeometric distribution within the R statistical environment. Annotation for every protein and protein family is available online: https://www.dropbox.com/sh/koq9oo0bumx4m19/AABavnbOn8zuqawvgAEiG90_a?dl=0

Signal peptides, and an additional 10 amino acids adjacent to the signal peptides, were extracted for every protein of those 92 gene families predicted to contain signal peptides. Signal peptides were aligned using a locally installed version of GLAM [43] using the single letter amino acid code as an alphabet. The best alignments were further examined for all 92 gene families to assess patterns of signal peptide divergence.

The extent of similarity between signal peptides within 94 protein families was assessed using the HH-suite 2.0.9 program [44, 45]. Alignments from GLAM were used to construct hidden Markov models with the hhmake program, and similarity between the models was determined by an all-versus-all search using the hhsearch program. E-values generated by hhsearch were used as measures of similarity in hierarchical clustering using the Ward algorithm within a custom R-script.

### Identification of genes functionally associated with secondary SecA_DEAD domain proteins

Sequences of the 10 proteins encoded up- and downstream of each secondary SecA_DEAD domain protein in every genome were retrieved and grouped into clusters using single linkage clustering, applying 25% amino acid identity and 60% alignment extension thresholds within the blastclust program. Several clusters of various sizes were identified. Gene neighbor scores from the STRING database were used to confirm functional association between the clusters [46]. Five clusters were analyzed further by performing phylogenetic and domain composition analysis.

### Reanalysis of expression data and regulatory sequence analysis

Raw microarray data for *Methylobacterium extorquens AM1* were downloaded from the GEO website (http://www.ncbi.nlm.nih.gov/geo/), series GSE42116 [47], and processed using the limma R package [48]. The normexp procedure [49] was used for background correction and the quantile method [50] was used for between-array normalization. Log2-transformed values of intensity for spots corresponding to genes of interest were extracted and plotted using R.

The *M*. *extorquens AM1* genome was analyzed to assess the presence of terminators of transcription using TransTermHP with default settings [51].

### Benchmarking gene neighbor scores from STRING

Gene neighbor scores were extracted for every pair of predicted orthologous groups from the COG.links.detailed.v9.1.txt file obtained from the STRING website (http://string-db.org). Correspondence between every protein in the *Escherichia coli* K-12 substr. MG1655 genome, and orthologous groups in STRING, was established using the COG.mappings.v9.1.txt file. Information about protein-protein functional association was extracted from the pathways.col and protcplxs.col files available from the EcoCyc version 18.5 database [52]. Correspondence between gene names used in EcoCyc and STRING was established using the NCBI taxonomy ID for the *E*. *coli* genome (511145), and BLATTNER-ID reported for every protein in the genes.col file from EcoCyc. We used proteins in complexes, pathways, and in both complexes and pathways as true sets of interacting proteins. To generate ROC curves we applied gene neighbor score threshold values ranging from 0 to 905 to classify every pair of proteins in the *E*. *coli* genome as interacting or non-interacting, and calculate sensitivity and (1-specificity) for every threshold value as follows:

Sensitivity=TP/(TP+FN)

1-Specificity=FP/(FP+TN)

Where TP—true positive, FP—false positive; TN—true negative; FN—false negative.

## Results and Discussion

### Signal peptides are over-represented in gene families associated with PVC intracellular membranes

The detection of gene families associated with PVC intracellular membranes, along with their predicted domain composition and signal peptide prediction, was reported in Kamneva *et al*, 2012 [26]. Some of these gene families contain predicted type I signal peptides. This prompted us to conduct a more systematic examination here to determine whether signal peptides were over-represented in proteins associated with the intracellular membranes. Prediction of signal peptides is still an open problem complicated by both the short length of the peptides and ambiguity in translation start site annotation [53], but there has been recent progress in developing applicable computational tools. Here we determined the presence of type I and II signal peptides using the lipoP program (similar results were obtained when the signalP program was used) and twin-arginine signal peptides using PFAM searches in every gene family from Kamneva *et al*, 2012 [26]. Subsequent statistical analysis (workflow shown in Figure A in S1 Fig) showed that compared to the genomic average, signal peptides were significantly over-represented in genes associated with endomembranes; 92 out of 149 of these gene families contained signal peptides, compared to 16,855 gene families out of 90,731 in the total dataset (p-value < 1e-16). When signalP-based classification was used we found that 59 out of 149 gene families associated with endomembranes had signal peptides compared to 9,984 gene families out of 90,731 in the total dataset (p-value < 1e-16). Fewer gene families were predicted to contain signal peptides with use of signalP because it is a much more conservative tool than lipoP. Twin-arginine signal peptides were also overrepresented in gene families associated with intracellular membranes, being found in four of these families, but in only 155 gene families in the entire set of 90,731 (p-value = 6.396e-6).

We also examined the amino acid composition of signal peptides present within proteins associated with PVC intracellular membranes and the protein region immediately adjacent to the signal peptide. It appeared that all protein families possessed peptide sequences similar to the canonical signal peptide or twin-arginine tag (S2 Fig). The structure of canonical signal peptides targeting proteins to Sec-dependent translocation has been extensively studied. The canonical signal peptide contains three domains: a positively charged N-terminal domain, followed by a hydrophobic core and a basic C-terminus [14]. The presence of canonical signal peptides suggests that the canonical Sec translocase is involved in targeting proteins preferentially present in intracellular membrane-bearing PVC bacteria. However, without further experimental work we cannot currently determine whether these proteins are targeted to the cytoplasmic membrane or to the intracellular membranes, or whether the canonical Sec-pathway is indeed involved in their targeting. If the canonical translocon is responsible for protein targeting to endomembranes, this suggests that PVC species and eukaryotic organisms possess similar mechanisms of intracellular membrane function which probably emerged independently. Protein sequences adjacent to signal peptides do not seem to include amino acids conserved across the gene families (S2 Fig).

### Domain composition and phylogenetic distribution of Sec pathway proteins across bacterial genomes

**Fig 1:**
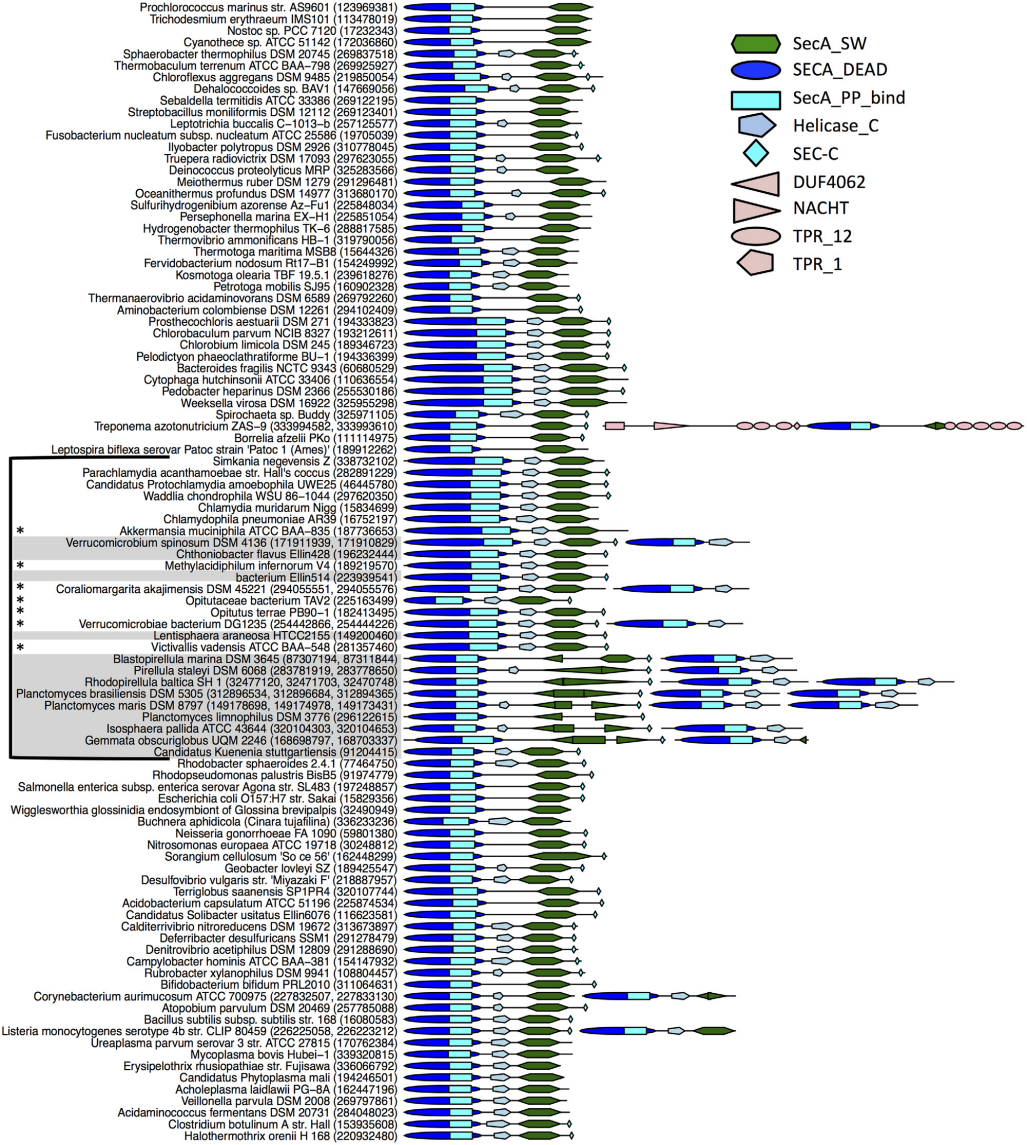
Domain architecture of SecA_DEAD domain-containing proteins in 99 genomes included in the analysis in Kamneva *et al*, 2012 [26]. Domain architecture of every protein containing SecA_DEAD domains is shown as identified using search against the Pfam [28] database; only domains showing hits above trusted cut-off are shown. Names of the organisms are shown on the left, grey background—organisms with intracellular membranes present, white background—absent, marked by * sign—organisms have not been examined using appropriate microscopy techniques. Gi numbers of corresponding proteins are shown in parentheses. Domain architecture of every identified protein is on the right, length of the protein is to scale, but distance between proteins is not. Names of the domains and shapes used to represent them are shown in the inset.

We further examined genes encoding Sec pathway components in order to identify evolutionary changes potentially associated with the presence of PVC intracellular membranes (workflow shown in Figure B in S1 Fig). Through domain analysis of Sec proteins encoded in the genomes of several model organisms, we identified marker domains for every Sec pathway component (S1 Table), and applied this information via a PFAM search to identify Sec proteins within 99 bacterial genomes in the dataset from Kamneva *et al*, 2012 [26]. Most genomes in the dataset encode the majority of Sec pathway components with very conserved domain composition and arrangement with no easily detectable changes (domain composition data are not shown but available online). In contrast, we determined that all members of the phylum *Planctomycetes* except *K*. *stuttgartiensis* have rearrangements affecting the SecA_SW domain in the canonical SecA protein. Such rearrangements were not observed in any other canonical SecA proteins in any other bacteria in the dataset (Fig 1). The scaffold domain of SecA_SW serves as a structural scaffold spanning the entire structure of the SecA ATPase [54], while the helical wing domain is implicated in the interaction between SecA and the SecYEG complex [55]. Therefore the observed domain rearrangements might affect folding of SecA as well as interaction with other components of the canonical translocase. However the phylogenetic pattern of SecA_SW domain rearrangement does not suggest its association with the presence of PVC intracellular membranes, as it was not observed in *Verrucomicrobia* and *Lentisphaerae* species, which are known to possess intracellular membranes. The phi correlation coefficient (an extension of the Pearson correlation coefficient for binary variables) between the rearrangement and patterns of presence/absence of intracellular membranes was 0.76.

Most genomes in the dataset were shown to encode a single copy of each predicted Sec pathway component, as has been shown before for other organisms, but the SecA ATPase signature domain SecA_DEAD was present within multiple proteins in some genomes (Fig 1). These included representative *Actinobacteria* and *Firmicutes* with previously reported SecA2 proteins known to function with the canonical Sec translocase (SecA2-only system) [56]. In some Gram-positive bacteria, both SecA and SecY are found in two copies and function within the SecA2-SecY2 system [56], but none of those genomes were present in our dataset [26]. In the case of the SecA2-SecY2 system, several co-located accessory proteins, unrelated to Sec components, have been identified. For a comprehensive review on SecA2 based systems see [56].

Additionally, we found that the genome of *Treponema azotonutricium* ZAS-9 (phylum *Spirochaetes*) also contains two proteins possessing SecA_DEAD and SecA_SW domains, one of which appears to have standard domain composition. The second copy contains additional tetratricopeptide repeat (TPR_12, TPR_1) domains forming tandem repeats, which are generally involved in protein-protein interactions [57]. This copy also features a NACHT domain, involved in signal transduction (apoptosis, transcription activation) in eukaryotes [58] and linked to metacaspase and various repeat domains in prokaryotes [59]. The presence of an additional SecA copy has not been reported previously for *Spirochaetes*. We further examined the domain architecture of NACHT-domain containing proteins in our dataset of 99 organisms (S2 Fig), and found that the genome of *T*. *azotonutricium* ZAS-9 encodes another protein (of unknown function) with DUF4062/NACHT/TPR domain architecture. This finding suggests a possible recombination/duplication event leading to emergence of the SecA_DEAD/SecA_SW domain-containing protein in the *T*. *azotonutricium* ZAS-9 genome. However, more detailed phylogenetic analysis is needed to address the question of evolutionary origin and relatedness of these proteins.

We also identified an additional SecA_DEAD domain protein encoded in the genomes of several PVC species, which is a novel finding. The secondary SecA_DEAD domain protein either lacks the SecA_SW domain entirely, or contains only a small fragment of it. These proteins are henceforth referred to as secondary SecA_DEAD domain proteins; this term provides more conservative annotation than SecA2, as involvement of these proteins with the Sec pathway has not been experimentally demonstrated. The presence of a secondary SecA_DEAD domain-containing protein in PVC bacteria is intriguing, but (as described above for SecA_SW domain rearrangements within canonical SecA proteins) the presence/absence profile of this protein is only weakly correlated with that of intracellular membranes (Phi correlation is 0.66). This suggests that the protein is not functionally related to PVC-specific intracellular membranes.

### Evolutionary origin of secondary SecA_DEAD domain proteins in PVC bacteria

The presence of a secondary SecA_DEAD domain protein in the genomes of several PVC bacteria is highly unusual, as the majority of bacterial genomes encode strictly one SecA_DEAD domain protein, SecA ATPase. This prompted us to investigate the evolutionary origin of these genes (workflow shown in Figure C in S1 Fig). We identified SecA_DEAD domain proteins encoded within representative distantly related bacterial genomes (extended genome dataset), and used these sequences for phylogenetic analysis. We used FastTree for phylogeny reconstruction, as its approximate likelihood computations allow much faster inference from large datasets, while performing comparably well with classical likelihood methods [35].

**Fig 2:**
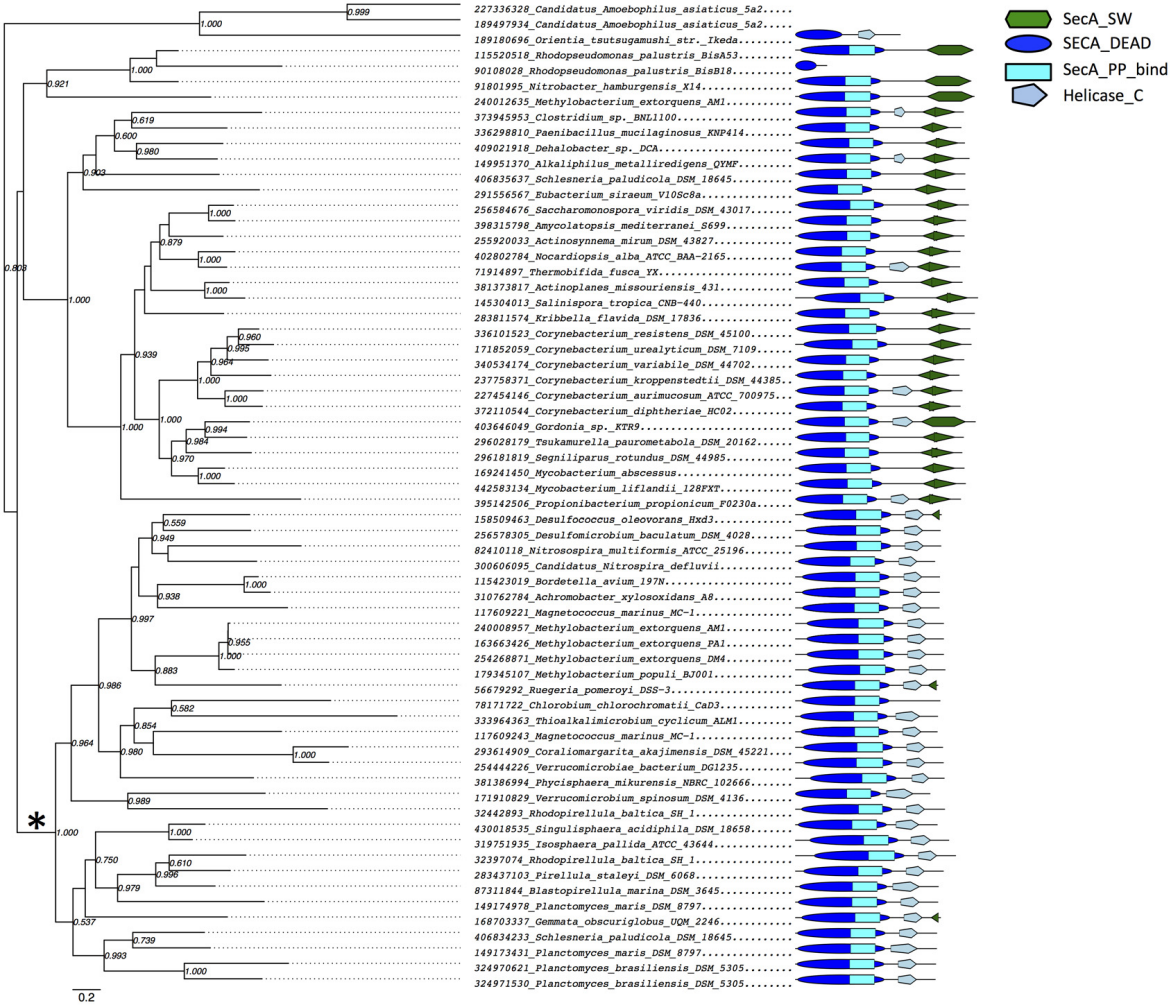
Phylogenetic relationship between SecA_DEAD domain proteins from PVC and other genomes. The clade of the SecA phylogeny containing additional SecA_DEAD domain proteins is shown. Phylogeny was recovered as described in Methods section. Bootstrap support values are shown if higher than 0.5. Domain composition of every protein is shown on the right except for three proteins belonging to the clade on the very top, which are omitted due to large size of the proteins, to maintain visual clarity. The lineage leading to a clade containing planctomycete, verrucomicrobial and proteobacterial sequences is marked by * sign.

The phylogeny obtained (Fig 2, S4 Fig) indicates that the secondary SecA_DEAD domain proteins from PVC bacteria are not related to the corresponding canonical SecA ATPase from those genomes, but rather to proteins with similar domain composition from several phyla of *Proteobacteria*, and one species each from the phyla *Chlorobi* and *Nitrospirae* (Fig 2, S4 Fig). This suggests that these proteins have been shared between organisms via horizontal gene transfer (HGT) or that they were present in the last common ancestor of those species and were subjected to extensive gene loss throughout the bacterial clade of the tree of life. Since the occurrence of secondary SecA_DEAD domain-containing proteins seems to be unrelated to the presence of PVC intracellular membranes, the presence of these genes in other bacterial clades should not be interpreted as evidence that these organisms possess intracellular membranes. It is noticeable that the proteins within this clade have lost their wing-scaffold domain (either completely or partially), indicating structural divergence of this protein and canonical SecA.

The clade containing PVC secondary SecA_DEAD domain proteins is related to the clade containing SecA2 proteins from *Actinobacteria*, again suggesting either ancient origin of these genes or that they have been transferred horizontally. In terms of domain composition, proteins in this clade seem to have undergone domain rearrangements in their wing-scaffold domain consistent with previous observations [56], but not to the extent of changes observed in the sister clade described above.

### Secondary SecA_DEAD domain proteins from *Planctomycetes*, *Verrucomicrobia* and other bacteria are encoded within a conserved genomic locus

In order to identify genes functionally associated with secondary SecA_DEAD domain proteins in PVC, *Proteobacteria*, *Nitrospirae* and *Chlorobi* we examined the genomic neighborhoods of every protein from a PVC-related clade (Fig 2; workflow shown in Figure D in S1 Fig). In order to determine whether there is a conserved neighborhood present around the secondary SecA_DEAD domain proteins, we extracted all relevant proteins and clustered their sequences using single linkage clustering based on protein sequence identity, using the blastclust program. We obtainined a number of clusters of different sizes (S5 Fig, S2 Table). The first three clusters are of particular interest because these proteins are encoded by a larger number of genomic neighborhoods around secondary SecA_DEAD domain proteins (S2 Table). Proteins in the first, second, and third clusters are annotated as peptidases, secretion proteins, and signal transduction/sensory proteins, respectively. Additionally, we examined relevant gene neighbor scores reported for every pair of orthologous groups in the STRING database [46]. The gene neighbor method is based on an empirical observation that functionally related genes tend to be encoded within genomic clusters in microbial genomes. The fact that functionally related genes are co-encoded is attributed to the inherent advantages of transcriptional co-regulation of genes located in close proximity within genomes [60] and by co-inheritance of functionally related genes in the event of HGT. The gene neighbor method has been shown to outperform other known genome content-based methods [61].

**Fig 3:**
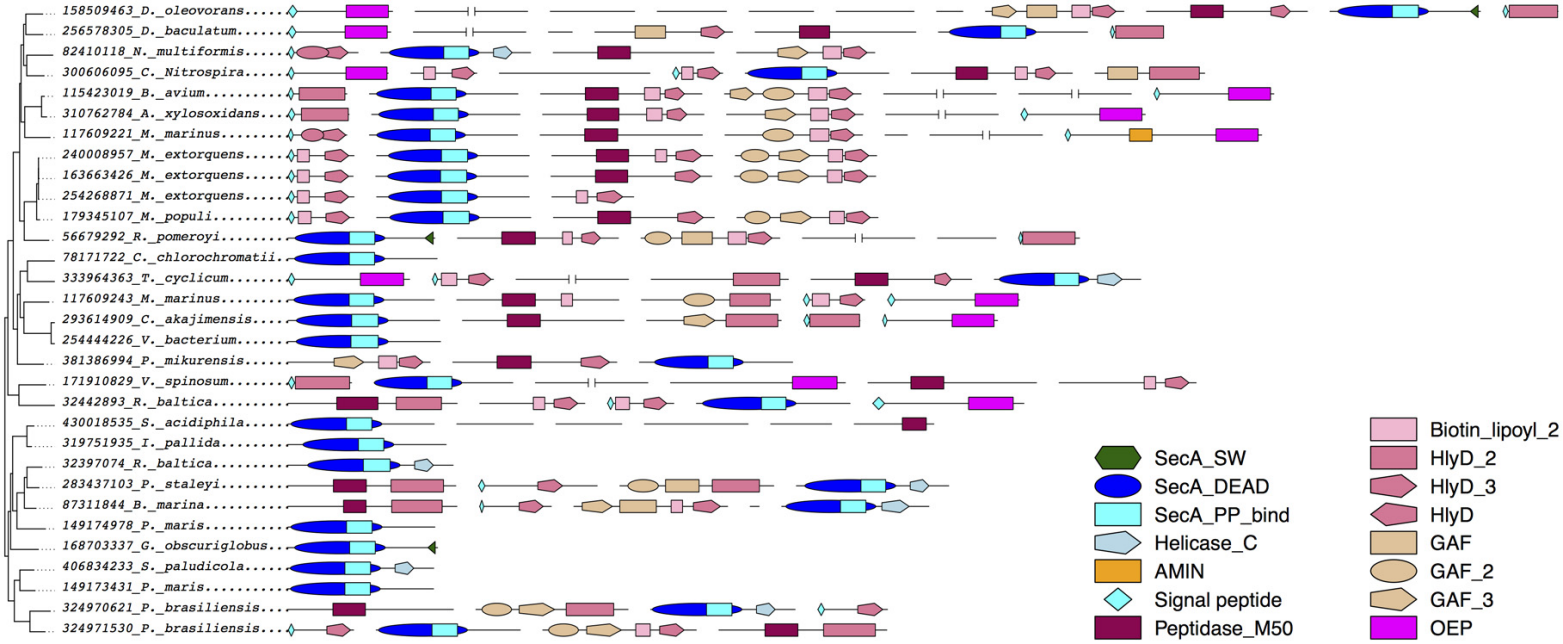
Genomic neighborhood of genes encoding secondary SecA_DEAD domain proteins from PVC and other genomes. Domain composition of proteins carrying domains present in several copies in close proximity to secondary SecA_DEAD domain proteins across the genomic loci are shown. Protein length is to scale unless pictured as an interrupted line, in which case protein had a length over 1,000 amino acids. Names of the domain shapes and different shading/filling used to represent them are shown on the right. Domains canonically related to the type I secretion system (different HlyD domains and OEP domain) are filled in white hexagons and pentagons or shown as hexagons with diagonal stripes.

We extracted gene neighbor scores for pairs of orthologous groups corresponding to SecA_DEAD domain proteins and proteins from clusters one, two and three (S3 Table). The gene neighbor scores for association between SecA homologs and other proteins tend to be smaller, which is likely attributed to the fact that this group includes canonical SecA as well as secondary SecA_DEAD domain proteins. The scores are still high and correspond to either the very beginning or the middle of the ROC curve when gene neighbor scores are tested on proteins from known pathways and complexes in the *Escherichia coli* str. K-12 substr. MG1655 genome, as reported in EcoCyc [52] (S6 Fig). Therefore, we conclude that secondary SecA_DEAD domain proteins are functionally associated with at least three other proteins and that they are often found within conserved genomic neighborhoods with those proteins. A schematic representation of the conserved genomic loci in various bacteria (Fig 3) shows only domains found in those secondary SecA_DEAD and proteins from the three clusters that were predicted to be functionally associated.

### The newly identified conserved genomic module encodes proteins related to the type I secretion system, alongside the secondary SecA_DEAD domain proteins

Conservation of the genomic neighborhood around secondary SecA2_DEAD domain proteins in PVC genomes and genomes of other bacteria suggests that these loci encode molecular machinery responsible for a distinct cellular process. We conducted domain analysis of proteins within the loci, in an attempt to predict the nature of this process. The proteins of the first, second, and third clusters contain different types of HlyD domains and also bear rather weak (but recognizable) similarity to the Peptidase_M50 domain (Fig 3). HlyD domains canonically function within the Type I secretion system (TISS). This secretion system acts on different kinds of substrates ranging from small molecules to polypeptides [62]. It is typically composed of three parts: an ABC-transporter (HlyB in *E*. *coli*), a membrane fusion protein (HlyD in *E*. *coli*), and an outer-membrane channel (TolC in *E*. *coli*). The ABC transporter provides the energy for the transport via ATP hydrolysis and a pore spanning the cytoplasmic membrane to allow passage of molecules. The characteristic domains of HlyB include different parts of ABC transporters. The membrane fusion protein forms a canal spanning the periplasmic space [63]. Different classes of HlyD and biotin_lipoyl domains can be considered as the signature domains of membrane fusion proteins. The last component of the system is an outer-membrane channel of the TolC family, which allows transport outside the cell and is characterized by the presence of OEP protein domains.

We identified proteins carrying numerous HlyD domains within the genomic neighborhood of secondary SecA_DEAD domain proteins (Fig 3). In some cases we also observed OEP domain proteins, which are normally encoded elsewhere in a genome but which are sometimes found adjacent to other TISS proteins [64]. While we could not identify homologs of an ABC transporter within these loci, the SecA_DEAD domain is responsible for ATP hydrolysis when functioning within the canonical SecA, therefore it seems possible that the secondary SecA_DEAD domain protein is co-opted here as a functional ATPase. However, while this protein may provide ATPase activity, it cannot form a trans-membrane pore and completely abolish the need for an ABC-transporter. The peptidase_M50 domain proteins encoded within the genomic loci are good candidates for transmembrane proteins; MEROPS peptidases are generally known to be buried within the lipid bilayer [65, 66]. Additionally, peptidase_M50 domain proteins from the conserved genomic loci are predicted to contain trans-membrane helices (S7 Fig), and therefore could potentially form a pore in the cytoplasmic membrane to complete required ABC transporter function.

Another protein present in the majority of the loci is a GAF domain-containing protein. GAF domains are canonically involved in signal transduction and are usually linked to some effector domain of enzymatic activity [67]. In this case, GAF domain proteins also contain HlyD and biotin_lipoyl domains; the function of this member of the conserved locus is unclear but the presence of the GAF domain suggests a regulatory and sensory role for this protein.

In order to evaluate the evolutionary history of additional proteins encoded within the conserved genomic loci, we extended five homologous groups and reconstructed the phylogenies of the extended gene families (S8, S9, S10, S11 and S12 Figs). It appears that genes from these genomic loci are related to sequences from *Planctomycetes*, *Verrucomicrobia*, different subdivisions of the *Proteobacteria* and a few species from other phyla, further supporting lateral transfer of this conserved locus between the genomes. In-depth phylogenetic and functional sequence analysis will be required to reveal the detailed evolutionary history of these additional components of the locus. From the extended phylogeny, it is also evident that while some of the secondary SecA_DEAD domain proteins are not co-encoded with functionally associated proteins, those proteins are found elsewhere in the genome (S13 Fig).

Although the presence of conserved domains in co-encoded proteins provides evidence for a secretion-related function of the conserved genomic locus, their broader biological role is completely unknown. Gene expression analysis, identifying changes in expression of genes within the locus in response to changes in the environment, could help to predict this biological role. The only organism possessing the newly identified locus and having available gene expression data is *Methylobacterium extorquens* AM1 [47]. In this genome, four genes MexAM1_META1p2402, -2401, -2400 and -2399 (coding for GAF, Peptidase_M50, SecA_DEAD, HlyD/Biotin_lipoyl domain proteins respectively; proteins from the three clusters described previously and secondary SecA_DEAD) are members of one operon (S4 Table) on the reverse DNA strand (data from microbesonline database [68, 69]). Genome-wide expression was measured in the exponential phase of growth of *M*. *extorquens* AM1 using microarray technology. Our reanalysis of this expression data set indicates that the ORF encoding the GAF domain containing protein (MexAM1_META1p2402) is expressed in the exponential growth phase, using for comparison the ATP synthase F1 sector operon genes recognized to be actively transcribed during exponential growth [70] (S14 Fig, S4 Table). This result, together with sequence analysis of the region, indicates the presence of an internal terminator within the operon (S5 Table) and further supports the regulatory role of this protein in *M*. *extorquens* AM1. The rest of the genes are either not expressed, or are expressed at a very low level, suggesting they do not play a role under the experimental conditions. Application of molecular and cell biology experimental approaches will be required to elucidate the molecular and cellular functions, as well as the intracellular localization, of proteins encoded by the conserved locus, and molecular complexes in which they may participate.

## Conclusions

In the present study, we examined the possibility that proteins preferentially present in intracellular membrane-bearing PVC bacteria are targeted to their cellular destination via the Sec pathway. We showed that canonical signal peptides are over-represented within those proteins, which implies involvement of a canonical translocase in their targeting. The recent development of genetic tools for species within *Planctomycetes* [71] and *Verrucomicrobia* [72] makes this hypothesis testable in the future using targeted mutagenesis, immunoelectron microscopy and other approaches requiring genetic tools.

We were not able to identify gene duplication, loss, or protein domain rearrangements affecting any of the canonical Sec proteins and statistically associated with the presence of intracellular membranes in PVC bacteria. However, we presented evidence for an additional SecA_DEAD domain protein within the genomes of several Gram-negative bacteria of the phyla *Planctomycetes*, *Verrucomicrobia*, *Proteobacteria* and only a few species from phyla *Chlorobi* and *Nitrospirae*. This, to the best of our knowledge, is the first case of a potentially duplicated SecA protein reported for Gram-negative bacteria. We also identified several proteins functionally associated with the additional SecA_DEAD domain proteins, often co-encoded with the additional SecA_DEAD domain proteins. These associated proteins contain domains specific for the Type I secretion system, which suggests a secretion-related function. Based on the results of our phylogenetic inference, it is likely that this conserved locus has been shared among different genomes via HGT.

## Supporting Information

S1 Fig Analysis workflow. (PDF)

S2 Fig Signal peptides found in proteins preferentially present in the intracellular membrane-bearing members of the PVC superphylum. Hierarchical clustering of signal peptides from 92 gene families is shown as a dendrogram. Logos of signal peptides are also shown for every gene family. Gene family numbers are indicated in the middle of the figure, starting with the letter X.
(PDF)

S3 Fig Domain architecture of NACHT domain-containing proteins encoded in 99 divergent bacterial genomes. Domain architecture of every protein containing a NACHT domain (identified by searching against the Pfam (28) database; only domains showing hits above trusted cut-off are shown). Numbers listed underneath organism names are gi numbers of protein sequences. *T*. *azotonitricum* ZAS-9 is highlighted in pink. Domain and protein length is to scale. A key to the domains is provided on the right.
(PDF)

S4 Fig Large-scale phylogeny of SecA_DEAD domain-containing proteins. The phylogenetic tree was reconstructed for all SecA_DEAD domain-containing proteins and visualized using the iTOL web-server. Branches of the tree are colored according to the bacterial phyla to which sequences belong; a key is provided on the left. The colored bars corresponding to the multi-phyla clade of SecA_DEAD domain-containing proteins explored further in Fig 2 are circled.
(PDF)

S5 Fig Distribution of sizes of protein sequence clusters encoded within genomic neighborhoods of SecA2 proteins from PVC and other bacterial genomes. Protein sequences corresponding to genes encoded within the neighborhood of SecA2 proteins were clustered. The cluster corresponding to SecA_DEAD domain-containing proteins is marked as “SecA2”. The presence in a number of genomes of similar sequences encoded in close proximity to SecA_DEAD domain-containing proteins indicates that those proteins might be functionally related. Bars corresponding to large clusters examined further are shaded.
(PDF)

S6 Fig ROC curve benchmarking the use of gene neighbor scores reported in STRING to predict functionally associated proteins. *E*. *coli* K-12 substr. MG1655 proteins reported in EcoCyc were used as a test set. Performance on three different true positive sets is shown in different colors as indicated on the figure. Points on the curves corresponding to different thresholds of gene neighbor scores are shown by blue marks indicated on the figure.
(PDF)

S7 Fig Transmembrane helix prediction in characterized and predicted HlyB proteins. Upper panel: Prediction for HlyB from the *E*. *coli* O157 H7 Sakai genome (identifier from KEGG database is shown). Lower panel: Prediction for hypothetical protein DSM3645_23885 from the *B*. *marina* DSM 3645 genome (gi number is shown as an identifier). Predictions were carried out and visualized using the TMHMM web-server (http://www.cbs.dtu.dk/services/TMHMM/).
(PDF)

S8 Fig Unrooted phylogeny and domain composition of Peptidase_M50 domain-containing proteins (Cluster 1). Unrooted phylogenetic tree was reconstructed for all homologs of proteins from cluster 1. Bootstrap values less than 0.5 are not shown. Scale bar at left represents protein evolutionary distance equivalent to 0.2 substitutions per amino acid site. Domain architecture was identified by searching against the Pfam (28) database. Numbers adjacent to the organism names represent protein sequence gi numbers. Red font indicates that the protein is located in close proximity to SecA_DEAD domain proteins. Domain and protein length is to scale. A key to the domains is provided on the right. Scale bar at right is equivalent to 100 amino acids of protein length. Blue vertical bar marks proteins used to generate S12 Fig.
(PDF)

S9 Fig Unrooted phylogeny and domain composition of putative secretion proteins (Cluster 2). Unrooted phylogenetic tree was reconstructed for all homologs of proteins from cluster 2. Bootstrap values less than 0.5 are not shown. Scale bar at left represents protein evolutionary distance equivalent to 0.2 substitutions per amino acid site. Domain architecture was identified by searching against the Pfam (28) database. Numbers adjacent to the organism names represent protein sequence gi numbers. Red font indicates that the protein is located in close proximity to SecA_DEAD domain proteins. Domain and protein length is to scale. A key to the domains is provided on the right. Scale bar at right is equivalent to 100 amino acids of protein length. Blue vertical bar marks proteins used to generate S12 Fig.
(PDF)

S10 Fig Unrooted phylogeny and domain composition of proteins from Cluster 3. The phylogenetic tree was reconstructed for all homologs of proteins from cluster 3. Bootstrap values less than 0.5 are not shown. Scale bar at left represents protein evolutionary distance equivalent to 0.2 substitutions per amino acid site. Domain architecture was identified by searching against the Pfam (28) database. Numbers adjacent to the organism names represent protein sequence gi numbers. Red font indicates that the protein is located in close proximity to SecA_DEAD domain proteins. Domain and protein length is to scale. A key to the domains is provided on the right. Scale bar at right is equivalent to 100 amino acids of protein length. Blue vertical bar marks proteins used to generate S12 Fig.
(PDF)

S11 Fig Unrooted phylogeny and domain composition of proteins from Cluster 4. The phylogenetic tree was reconstructed for all homologs of proteins from cluster 4. Bootstrap values less than 0.5 are not shown. Scale bar at left represents protein evolutionary distance equivalent to 0.2 substitutions per amino acid site. Domain architecture was identified by searching against the Pfam (28) database. Numbers adjacent to the organism names represent protein sequence gi numbers. Red font indicates that the protein is located in close proximity to SecA_DEAD domain proteins. Domain and protein length is to scale. A key to the domains is provided on the right. Scale bar at right is equivalent to 100 amino acids of protein length.
(PDF)

S12 Fig Unrooted phylogeny and domain composition of proteins from Cluster 5. The phylogenetic tree was reconstructed for all homologs of proteins from cluster 5. Bootstrap values less than 0.5 are not shown. Scale bar at left represents protein evolutionary distance equivalent to 0.2 substitutions per amino acid site. Domain architecture was identified by searching against the Pfam (28) database. Numbers adjacent to the organism names represent protein sequence gi numbers. Red font indicates that the protein is located in close proximity to SecA_DEAD domain proteins. Domain and protein length is to scale. A key to the domains is provided on the right. Scale bar at right is equivalent to 100 amino acids of protein length.
(PDF)

S13 Fig Distribution of genes from loci containing SecA_DEAD domain proteins across genomes. The heatmap visualizes the distribution of genes from loci containing SecA_DEAD domain proteins across various genomes. Red indicates presence of the gene near to the secondary SecA_DEAD domain protein, pink indicates presence of the gene elsewhere in the genome, blue indicates absence of the gene. Genome names are shown on the right, Phylum or class level taxonomic names are indicated in parentheses as follows: A—*Alphaproteobacteria;* B—*Betaproteobacteria;* G—*Gammaproteobacteria;* D/E—*delta/epsilon* subdivisions of the *Proteobacteria;* C—*Chlorobi;* N—*Nitrospirae;* P—*Planctomycetes;* V—*Verrucomicrobia*.
(PDF)

S14 Fig mRNA expression for ORFs in M. extorquens AM1. Expression level (log2-transformed intensity values for the 5’ and C1 microarray probes) of four ORFs encoded within genomic loci associated with SecA_DEAD domain-proteins (A), as well as of ATP synthase F1 genes recognized as housekeeping genes (B), are shown for several substrains of *M*. *extorquens* AM1. mRNA level was measured in three biological replicates and mean intensity value plotted as a dot; standard error is shown as an error bar for each point. Strains of *M*. *extorquens* AM1 used for these comparisons are described in [43] and designated by different symbols, including wild-type (○), mutant (●) and adapted mutant strains (□, ■, △, ▽, ×, *, ◇, ▲) as shown in the bottom of each panel. mRNA level was measured in the exponential growth phase. Four SecA_DEAD domain-associated genes are designated as MexAM1_META1p2402- MexAM1_META1p2399 and shown in part A of the figure. ORFs from the ATPase F1 encoding-operon (MexAM1_META1p1359- MexAM1_META1p1362) are shown in part B. Genomic locations for the start of every ORF, and the middle of every microarray probe, are shown above the map in black and grey, respectively.
(PDF)

S1 Table Inferred signature domains of Sec proteins. (PDF)

S2 Table Similar proteins encoded within genomic neighborhoods of SecA_DEAD domain proteins. *—protein sequence cluster; **—distance from SecA_DEAD protein; A—*Alphaproteobacteria;* B—*Betaproteobacteria;* G—*Gammaproteobacteria;* D/E—*Delta/Epsilon* subdivision of the *Proteobacteria;* N—*Nitrospirae;* P—*Planctomycetes;* V—*Verrucomicrobia;* Ph—phylum; Cl—Class.
(PDF)

S3 Table Gene neighbor scores for relevant pairs of orthologous groups from STRING. (PDF)

S4 Table Operon predictions for selected ORFs in the *M*. *extorquens* AM1 genome. (PDF)

S5 Table Transcription terminators in the MexAM1_META1p2399—MexAM1_META1p2402 region of the *M*. *extorquens* AM1 genome. (PDF)
