# Supplementary figures and images for: Proteins Related to the Type I Secretion System Are Associated with Secondary SecA_DEAD Domain Proteins in Some Species of Planctomycetes, Verrucomicrobia, Proteobacteria, Nitrospirae and Chlorobi
Source: PLoS One. 2015 Jun 1;10(6):e0129066. doi: 10.1371/journal.pone.0129066 (PMC4452313; doi:10.1371/journal.pone.0129066)

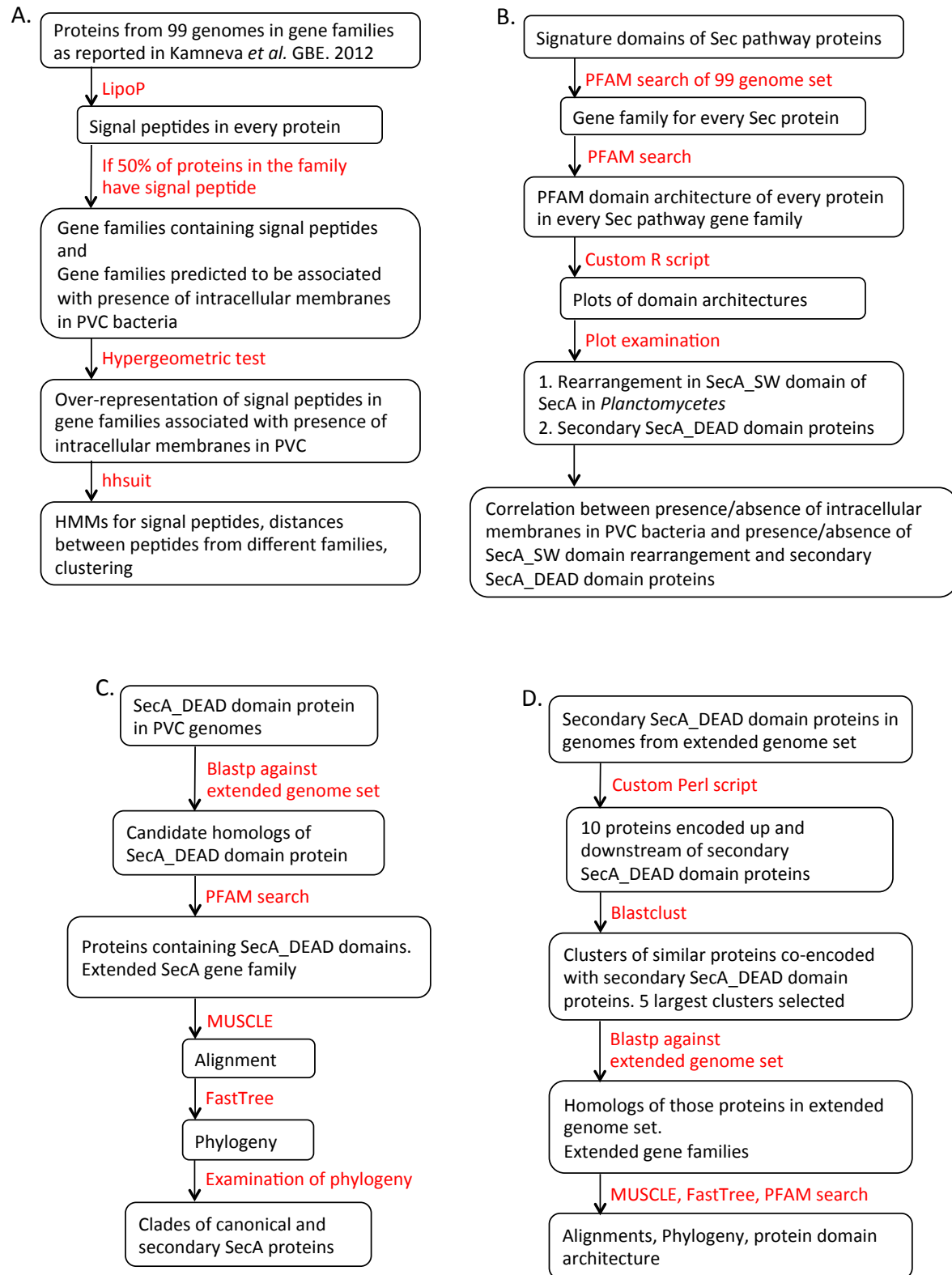

Supplement: S1 Fig — (PDF) [file pone.0129066.s001.pdf]

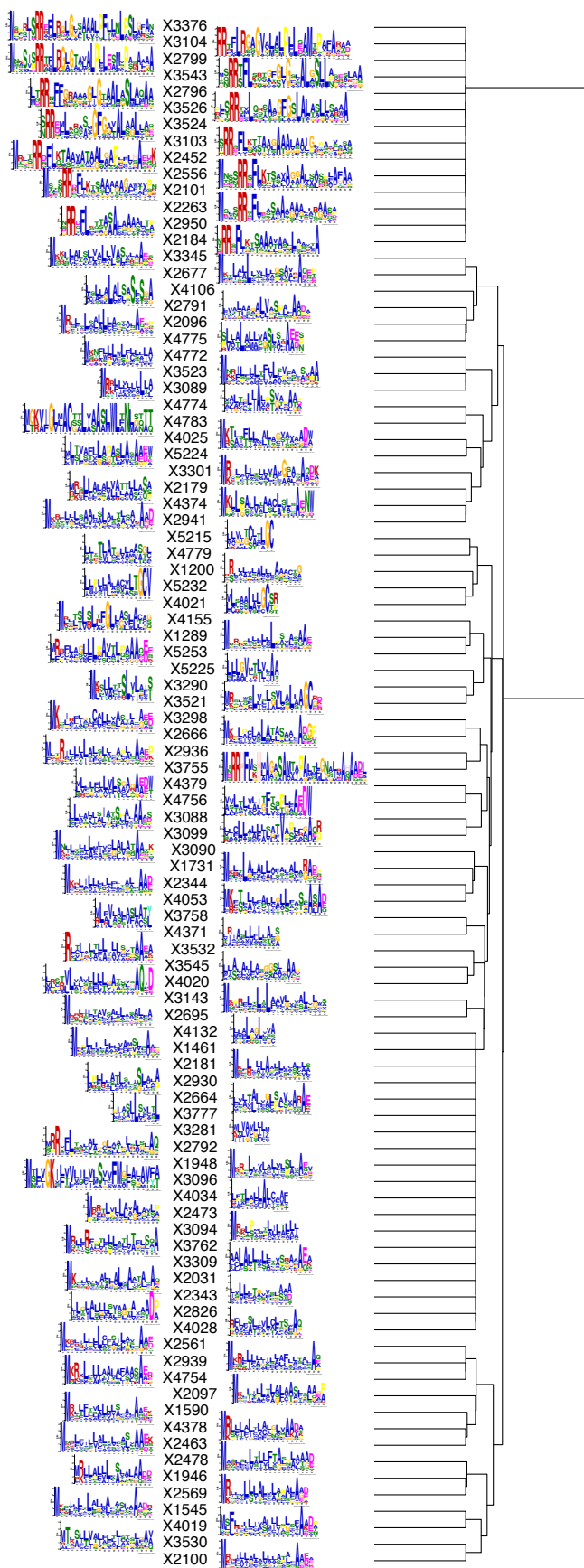

Supplement: S2 Fig — Hierarchical clustering of signal peptides from 92 gene families is shown as a dendrogram. Logos of signal peptides are also shown for every gene family. Gene family numbers are indicated in the middle of the figure, starting with the letter X. (PDF) [file pone.0129066.s002.pdf]

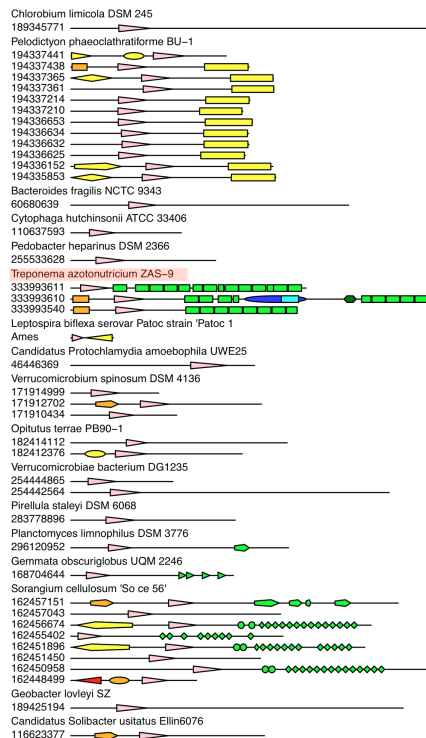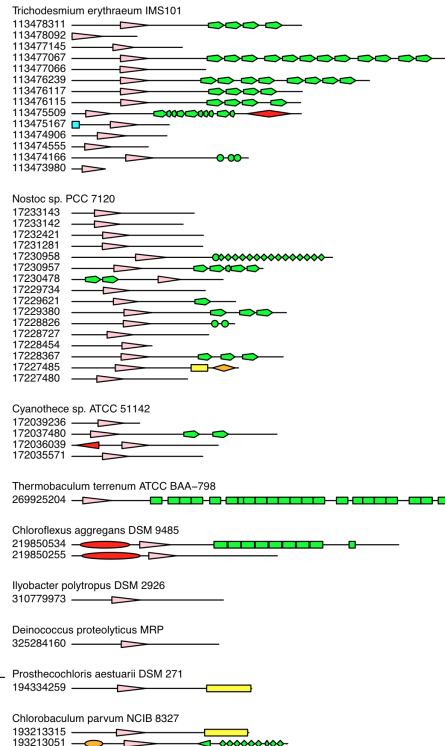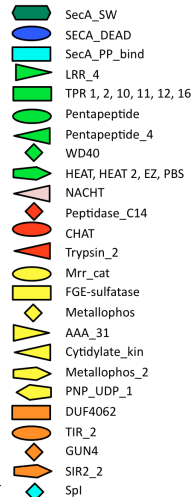

Supplement: S3 Fig — Domain architecture of every protein containing a NACHT domain (identified by searching against the Pfam (28) database; only domains showing hits above trusted cut-off are shown). Numbers listed underneath organism names are gi numbers of protein sequences. T. azotonitricum ZAS-9 is highlighted in pink. Domain and protein length is to scale. A key to the domains is provided on the right. (PDF) [file pone.0129066.s003.pdf]

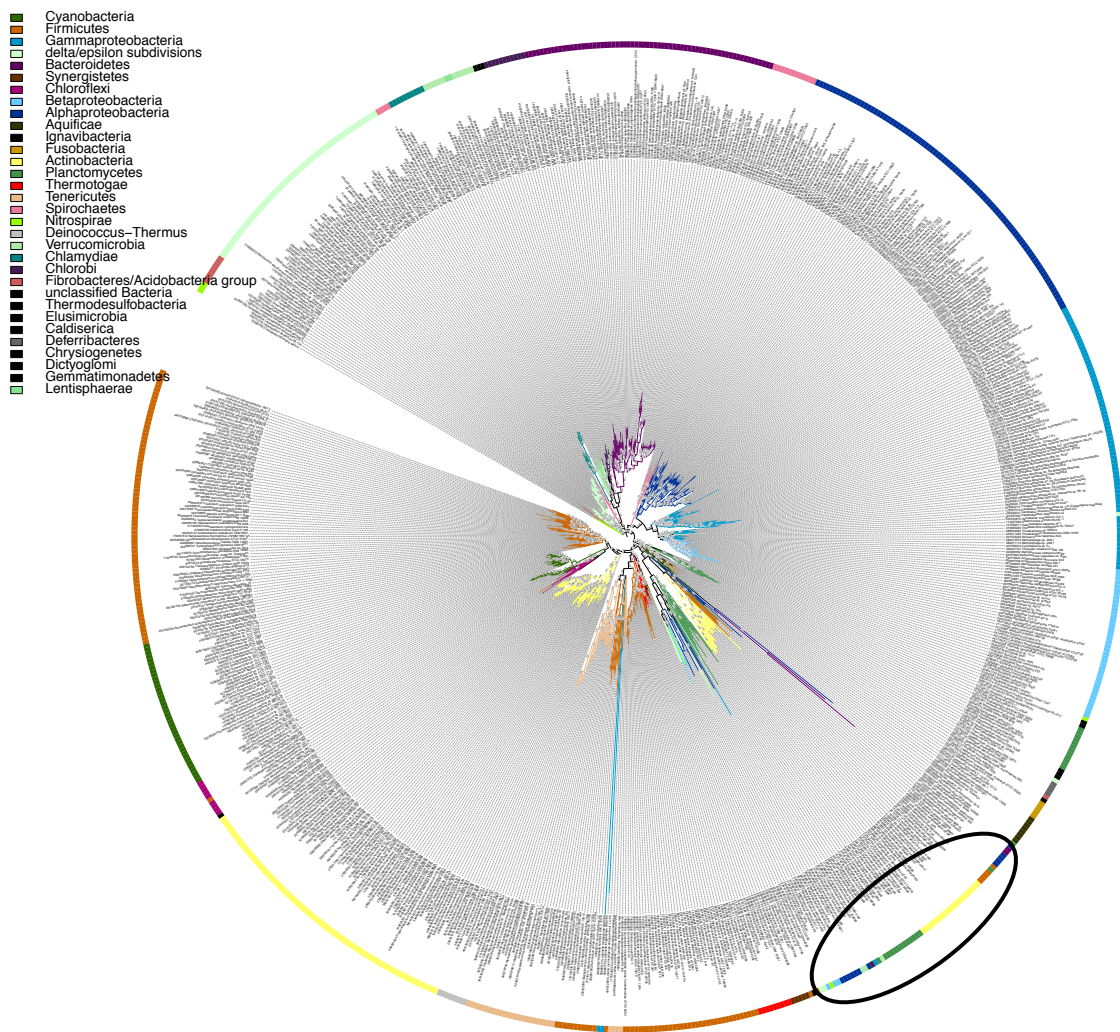

Supplement: S4 Fig — The phylogenetic tree was reconstructed for all SecA_DEAD domain-containing proteins and visualized using the iTOL web-server. Branches of the tree are colored according to the bacterial phyla to which sequences belong; a key is provided on the left. The colored bars corresponding to the multi-phyla clade of SecA_DEAD domain-containing proteins explored further in Fig 2 are circled. (PDF) [file pone.0129066.s004.pdf]

Size of sequence clusters

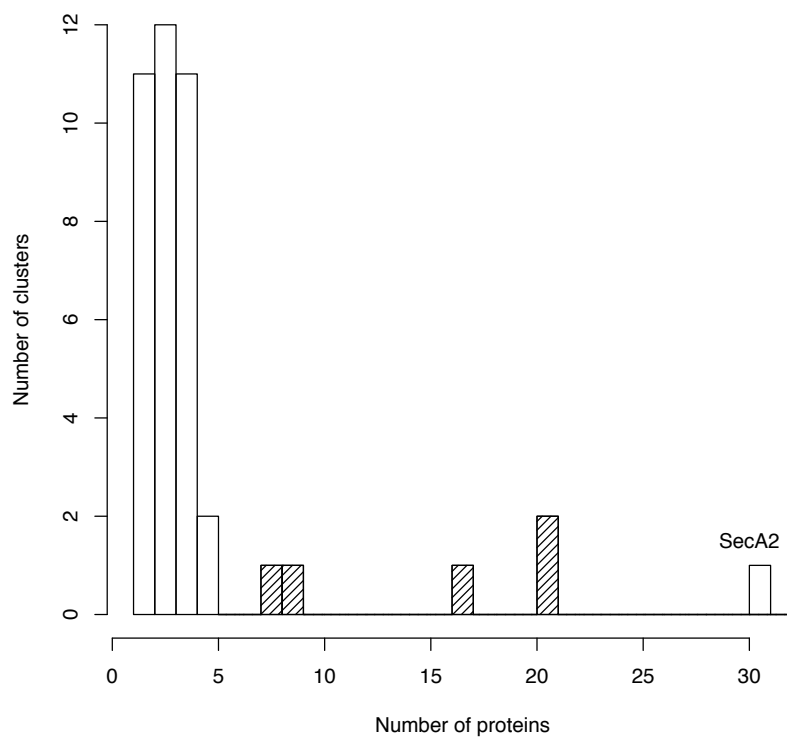

Supplement: S5 Fig — Protein sequences corresponding to genes encoded within the neighborhood of SecA2 proteins were clustered. The cluster corresponding to SecA_DEAD domain-containing proteins is marked as “SecA2”. The presence in a number of genomes of similar sequences encoded in close proximity to SecA_DEAD domain-containing proteins indicates that those proteins might be functionally related. Bars corresponding to large clusters examined further are shaded. (PDF) [file pone.0129066.s005.pdf]

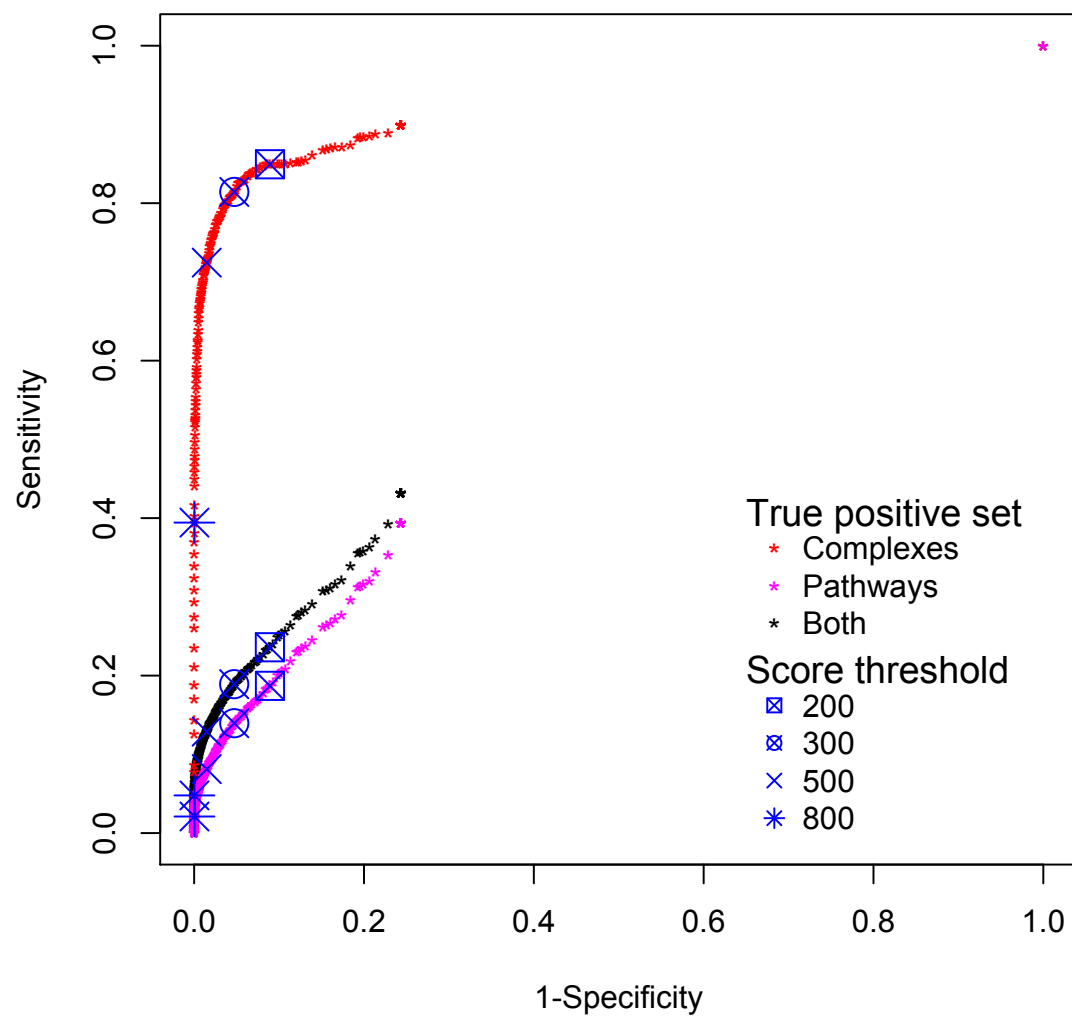

Supplement: S6 Fig — E. coli K-12 substr. MG1655 proteins reported in EcoCyc were used as a test set. Performance on three different true positive sets is shown in different colors as indicated on the figure. Points on the curves corresponding to different thresholds of gene neighbor scores are shown by blue marks indicated on the figure. (PDF) [file pone.0129066.s006.pdf]

ecs:pO157p19 hlyB

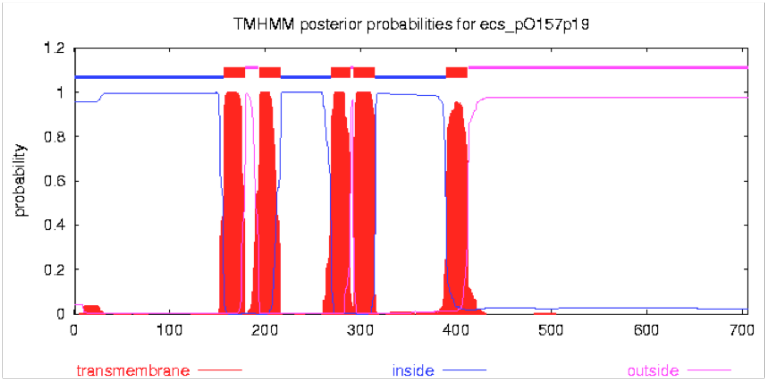

gi: 87311840 B. marina

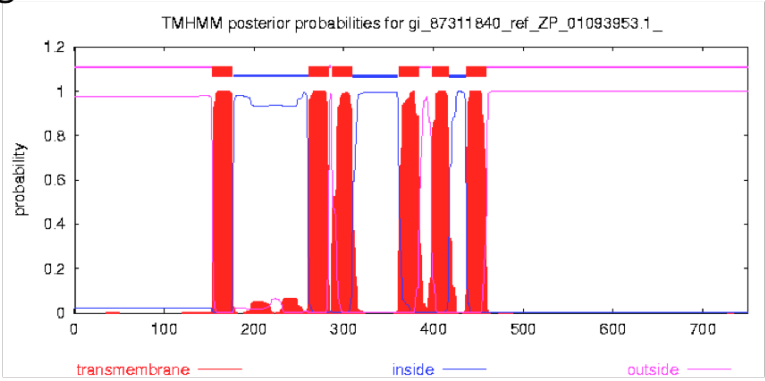

Supplement: S7 Fig — Upper panel: Prediction for HlyB from the E. coli O157 H7 Sakai genome (identifier from KEGG database is shown). Lower panel: Prediction for hypothetical protein DSM3645_23885 from the B. marina DSM 3645 genome (gi number is shown as an identifier). Predictions were carried out and visualized using the TMHMM web-server (http://www.cbs.dtu.dk/services/TMHMM/). (PDF) [file pone.0129066.s007.pdf]

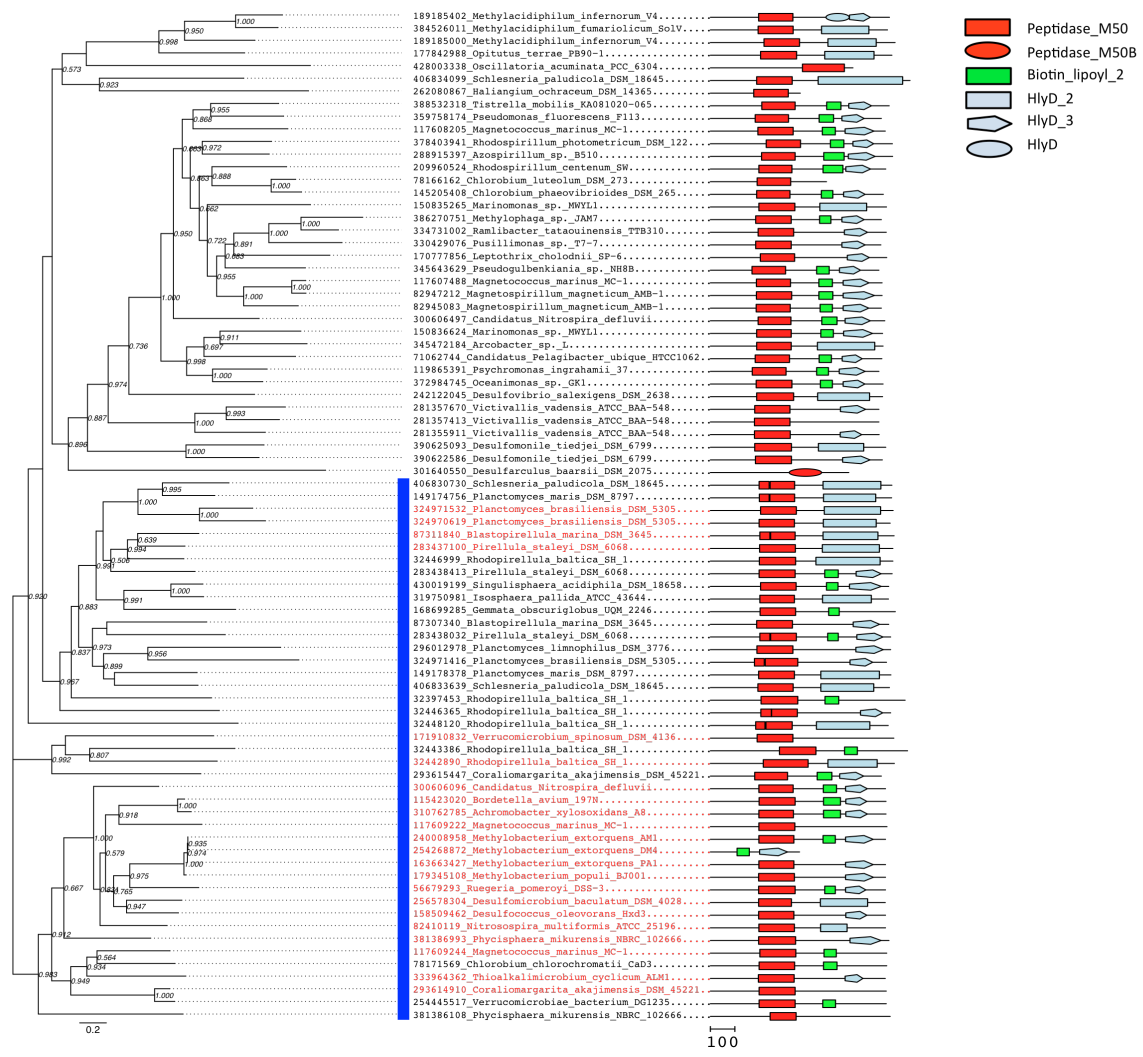

Supplement: S8 Fig — Unrooted phylogenetic tree was reconstructed for all homologs of proteins from cluster 1. Bootstrap values less than 0.5 are not shown. Scale bar at left represents protein evolutionary distance equivalent to 0.2 substitutions per amino acid site. Domain architecture was identified by searching against the Pfam (28) database. Numbers adjacent to the organism names represent protein sequence gi numbers. Red font indicates that the protein is located in close proximity to SecA_DEAD domain proteins. Domain and protein length is to scale. A key to the domains is provided on the right. Scale bar at right is equivalent to 100 amino acids of protein length. Blue vertical bar marks proteins used to generate S12 Fig. (PDF) [file pone.0129066.s008.pdf]

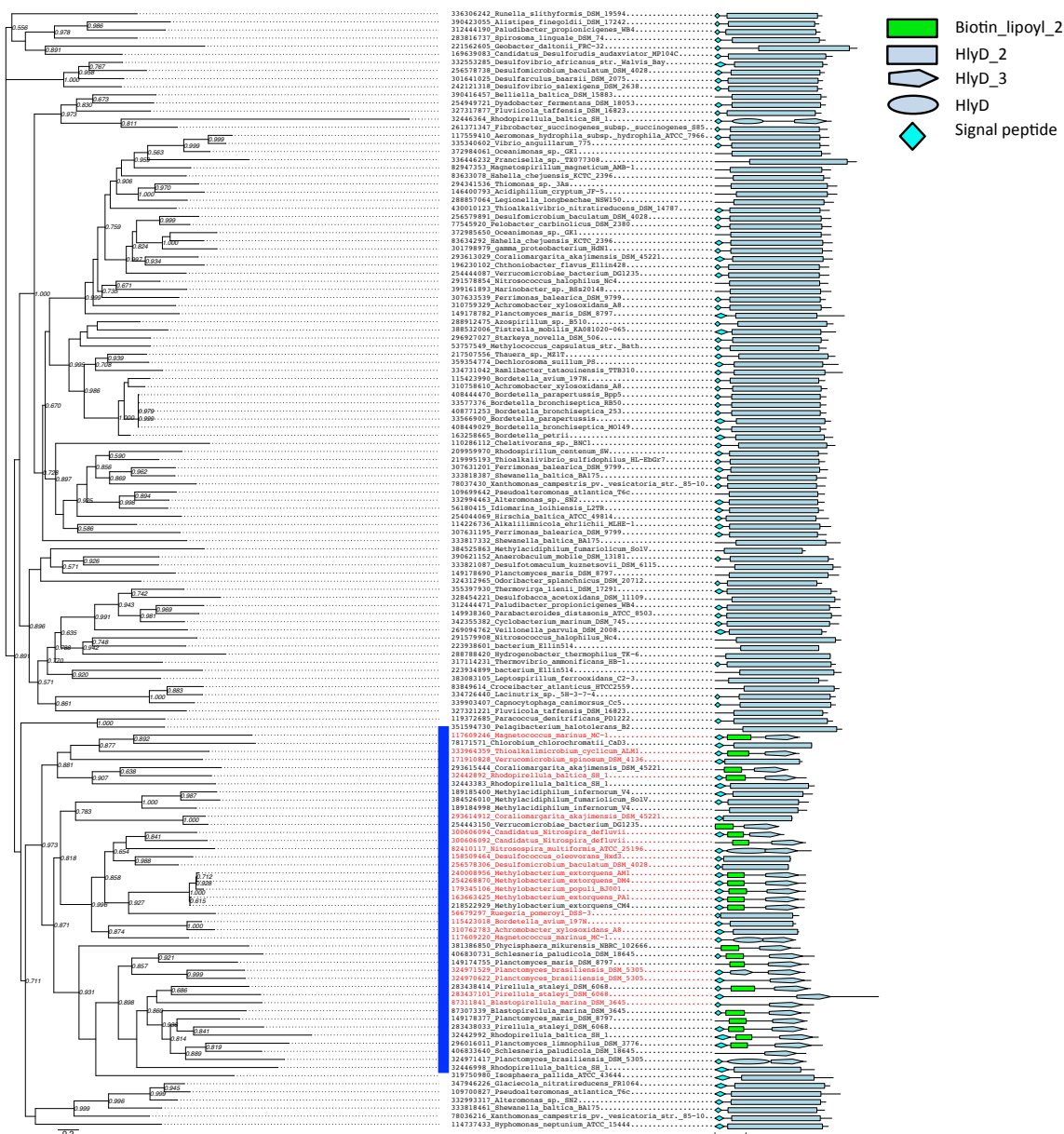

Supplement: S9 Fig — Unrooted phylogenetic tree was reconstructed for all homologs of proteins from cluster 2. Bootstrap values less than 0.5 are not shown. Scale bar at left represents protein evolutionary distance equivalent to 0.2 substitutions per amino acid site. Domain architecture was identified by searching against the Pfam (28) database. Numbers adjacent to the organism names represent protein sequence gi numbers. Red font indicates that the protein is located in close proximity to SecA_DEAD domain proteins. Domain and protein length is to scale. A key to the domains is provided on the right. Scale bar at right is equivalent to 100 amino acids of protein length. Blue vertical bar marks proteins used to generate S12 Fig. (PDF) [file pone.0129066.s009.pdf]

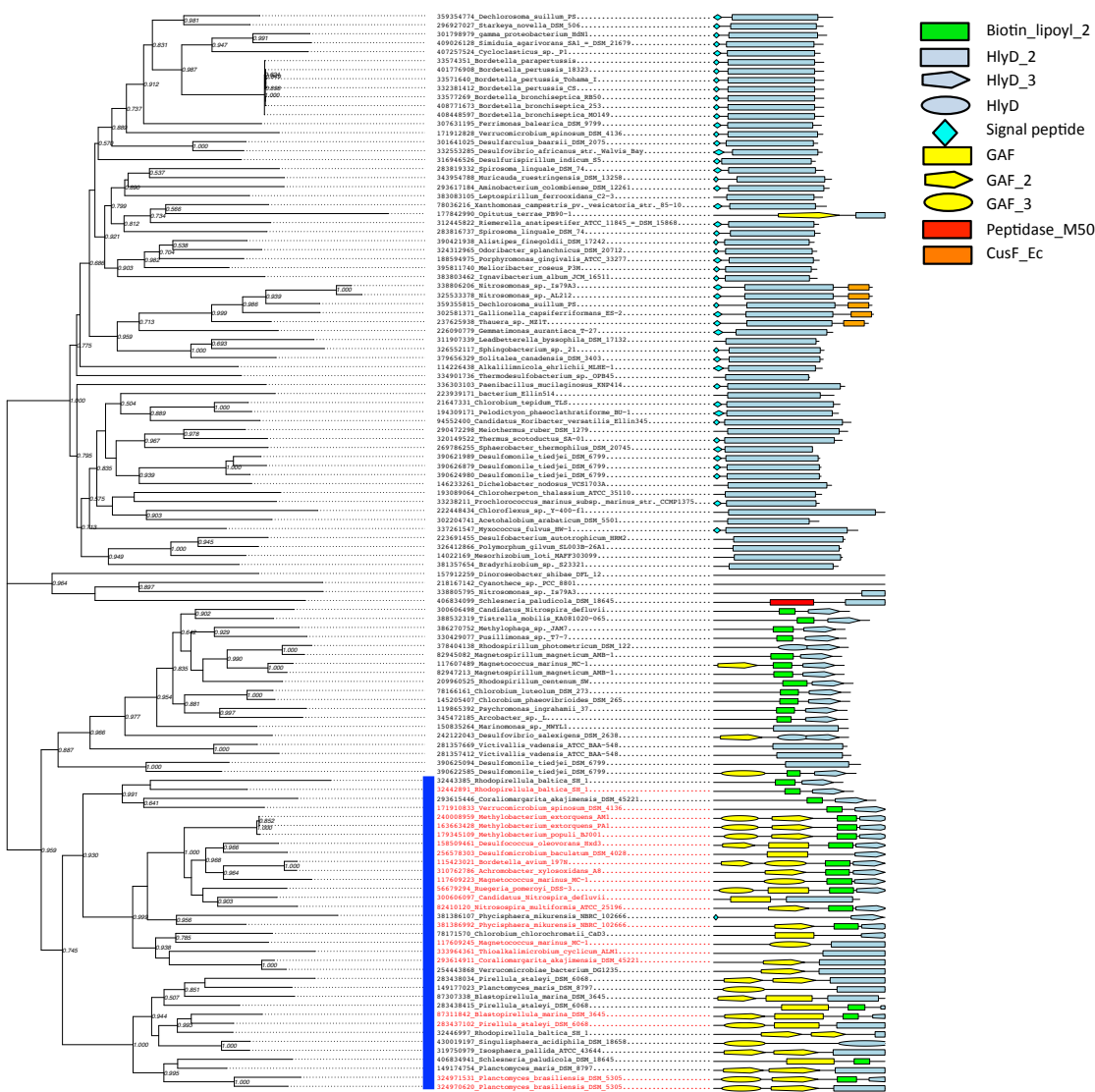

Supplement: S10 Fig — The phylogenetic tree was reconstructed for all homologs of proteins from cluster 3. Bootstrap values less than 0.5 are not shown. Scale bar at left represents protein evolutionary distance equivalent to 0.2 substitutions per amino acid site. Domain architecture was identified by searching against the Pfam (28) database. Numbers adjacent to the organism names represent protein sequence gi numbers. Red font indicates that the protein is located in close proximity to SecA_DEAD domain proteins. Domain and protein length is to scale. A key to the domains is provided on the right. Scale bar at right is equivalent to 100 amino acids of protein length. Blue vertical bar marks proteins used to generate S12 Fig. (PDF) [file pone.0129066.s010.pdf]

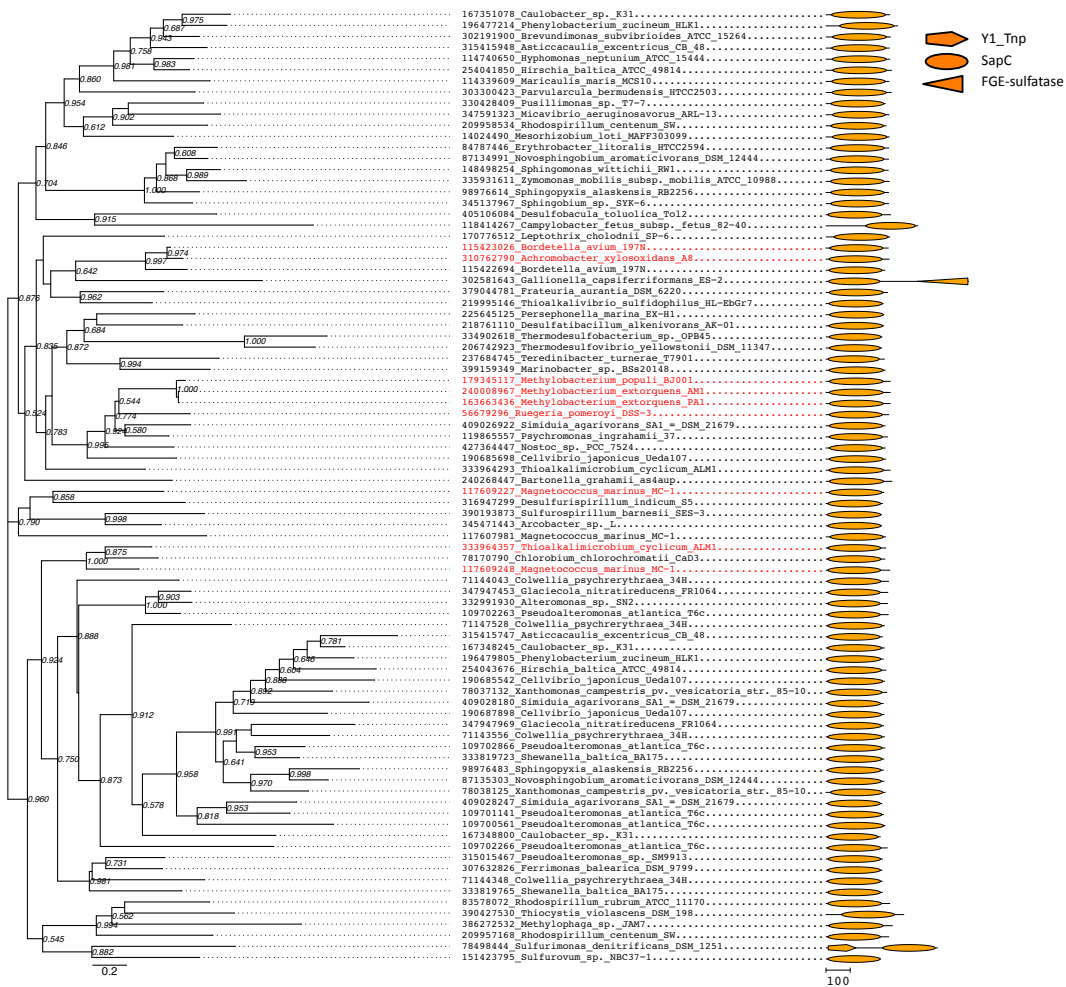

Supplement: S11 Fig — The phylogenetic tree was reconstructed for all homologs of proteins from cluster 4. Bootstrap values less than 0.5 are not shown. Scale bar at left represents protein evolutionary distance equivalent to 0.2 substitutions per amino acid site. Domain architecture was identified by searching against the Pfam (28) database. Numbers adjacent to the organism names represent protein sequence gi numbers. Red font indicates that the protein is located in close proximity to SecA_DEAD domain proteins. Domain and protein length is to scale. A key to the domains is provided on the right. Scale bar at right is equivalent to 100 amino acids of protein length. (PDF) [file pone.0129066.s011.pdf]

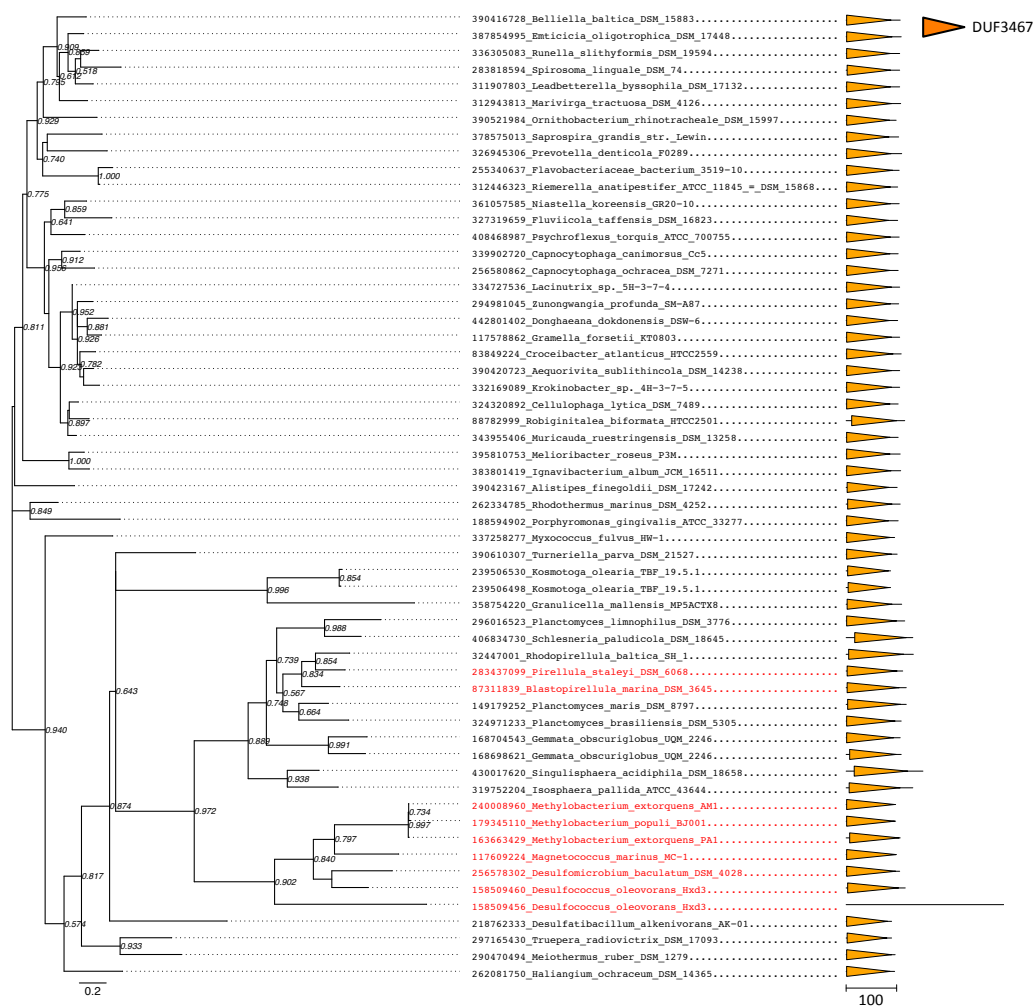

Supplement: S12 Fig — The phylogenetic tree was reconstructed for all homologs of proteins from cluster 5. Bootstrap values less than 0.5 are not shown. Scale bar at left represents protein evolutionary distance equivalent to 0.2 substitutions per amino acid site. Domain architecture was identified by searching against the Pfam (28) database. Numbers adjacent to the organism names represent protein sequence gi numbers. Red font indicates that the protein is located in close proximity to SecA_DEAD domain proteins. Domain and protein length is to scale. A key to the domains is provided on the right. Scale bar at right is equivalent to 100 amino acids of protein length. (PDF) [file pone.0129066.s012.pdf]

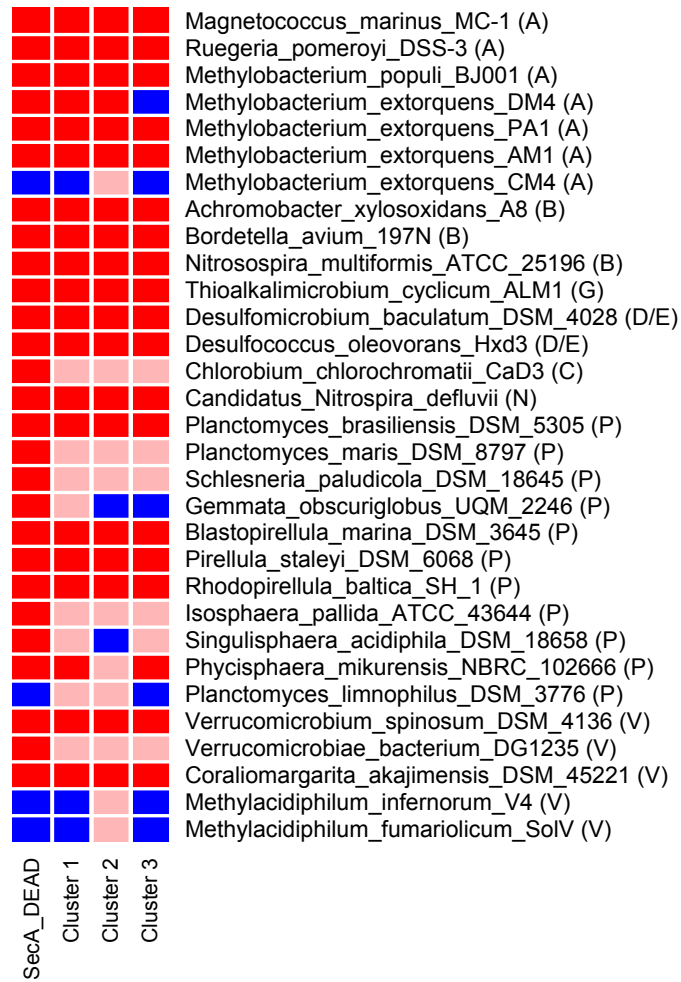

Supplement: S13 Fig — The heatmap visualizes the distribution of genes from loci containing SecA_DEAD domain proteins across various genomes. Red indicates presence of the gene near to the secondary SecA_DEAD domain protein, pink indicates presence of the gene elsewhere in the genome, blue indicates absence of the gene. Genome names are shown on the right, Phylum or class level taxonomic names are indicated in parentheses as follows: A—Alphaproteobacteria; B—Betaproteobacteria; G—Gammaproteobacteria; D/E—delta/epsilon subdivisions of the Proteobacteria; C—Chlorobi; N—Nitrospirae; P—Planctomycetes; V—Verrucomicrobia. (PDF) [file pone.0129066.s013.pdf]

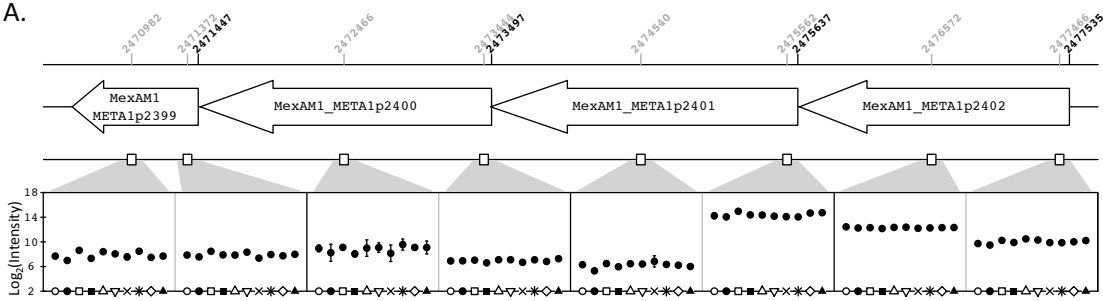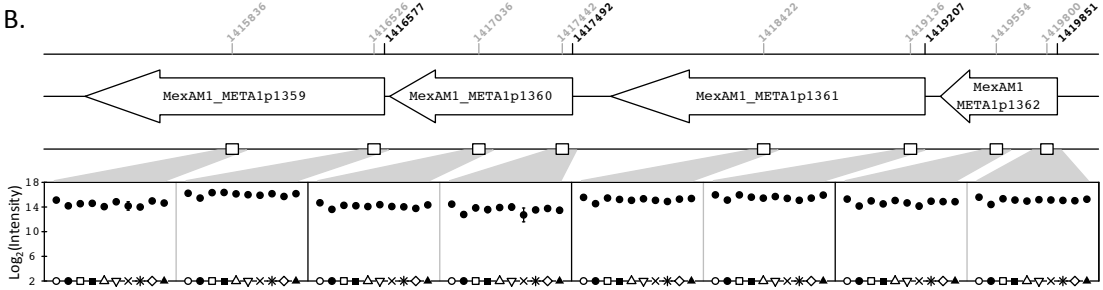

Supplement: S14 Fig — Expression level (log2-transformed intensity values for the 5’ and C1 microarray probes) of four ORFs encoded within genomic loci associated with SecA_DEAD domain-proteins (A), as well as of ATP synthase F1 genes recognized as housekeeping genes (B), are shown for several substrains of M. extorquens AM1. mRNA level was measured in three biological replicates and mean intensity value plotted as a dot; standard error is shown as an error bar for each point. Strains of M. extorquens AM1 used for these comparisons are described in [43] and designated by different symbols, including wild-type (○), mutant (●) and adapted mutant strains (□, ■, △, ▽, ×, *, ◇, ▲) as shown in the bottom of each panel. mRNA level was measured in the exponential growth phase. Four SecA_DEAD domain-associated genes are designated as MexAM1_META1p2402- MexAM1_META1p2399 and shown in part A of the figure. ORFs from the ATPase F1 encoding-operon (MexAM1_META1p1359- MexAM1_META1p1362) are shown in part B. Genomic locations for the start of every ORF, and the middle of every microarray probe, are shown above the map in black and grey, respectively. (PDF) [file pone.0129066.s014.pdf]
