# Supplementary material for: Proteins Related to the Type I Secretion System Are Associated with Secondary SecA_DEAD Domain Proteins in Some Species of Planctomycetes, Verrucomicrobia, Proteobacteria, Nitrospirae and Chlorobi
Source: PLoS One. 2015 Jun 1;10(6):e0129066. doi: 10.1371/journal.pone.0129066 (PMC4452313; doi:10.1371/journal.pone.0129066)
Supplement: S1 Table — (PDF) [file pone.0129066.s015.pdf]

	Nostoc	*Bifidobacterium*	*Bacillus*	*Escherichia*	*Chlamydia*	*Bacteroides*	Signature domain
**SecY**	SecY_80_419<br>2.50E-105	SecY_73_419<br>2.20E-103	SecY_68_414<br>1.00E-109	SecY_76_417<br>4.50E-121	SecY_73_433<br>2.10E-105	SecY_74_416<br>8.10E-107	SecY
**SecE**	SecE_18_73<br>8.30E-18	SecE_19_74<br>5.10E-15	SecE_3_58<br>2.30E-21	SecE_68_122<br>2.40E-21	SecE_21_73<br>3.70E-09	SecE_3_59<br>3.30E-17	SecE
**SecG**	SecG_5_73<br>4.40E-20	SecG_6_77<br>6.50E-20	SecG_3_75<br>1.10E-26	SecG_3_75<br>1.00E-26	SecG_6_75<br>5.00E-18	SecG_2_71<br>7.80E-20	SecG
**SecD/F**	Sec_GG_23_47<br>2.40E-06<br>SecD_SecF_275_439<br>8.40E-16		Sec_GG_23_51<br>1.60E-05<br>SecD_SecF_240_409<br>5.10E-17<br>Sec_GG_471_495<br>7.70E-05<br>SecD_SecF_543_727<br>5.30E-67	SecD-TM1_2_103<br>8.80E-38<br>Sec_GG_112_141<br>1.00E-07<br>SecD_SecF_434_602<br>1.40E-15	Sec_GG_569_595<br>0.016<br>SecD_SecF_887_1057<br>3.60E-17<br>SecD_SecF_1211_1392<br>7.10E-65	Sec_GG_73_94<br>6.60E-05<br>SecD_SecF_488_655<br>2.50E-19<br>Sec_GG_707_732<br>0.0015<br>SecD_SecF_812_994<br>5.50E-54	SecD_SecF
**YajC**		YajC_11_87<br>2.20E-17	YajC_4_84<br>1.60E-33	YajC_20_102<br>4.50E-28	YajC_36_109<br>8.20E-20	YajC_20_95<br>6.80E-31	YajC
**YidC**	60KD_IMP_25_344<br>4.50E-60	60KD_IMP_23_241<br>3.40E-46	60KD_IMP_62_259<br>4.60E-54	60KD_IMP_352_533<br>3.40E-67	60KD_IMP_568_771<br>5.00E-59	60KD_IMP_366_568<br>1.60E-54	60KD_IMP
**SecA**	SecA_DEAD_5_378<br>1.30E-121<br>SecA_PP_bind_227_335<br>2.60E-39<br>SecA_SW_703_917<br>4.40E-71	SecA_DEAD_13_383<br>4.40E-121<br>SecA_PP_bind_233_340<br>3.00E-37<br>SecA_SW_613_830<br>2.40E-71<br>SEC-C_940_957<br>3.10E-08	SecA_DEAD_7_382<br>5.10E-118<br>SecA_PP_bind_228_338<br>8.70E-43<br>Helicase_C_447_530<br>0.00041<br>SecA_SW_568_780<br>3.40E-69<br>SEC-C_821_839<br>3.00E-09	SecA_DEAD_7_401<br>1.50E-121<br>SecA_PP_bind_229_358<br>4.90E-40<br>SecA_SW_617_830<br>4.00E-76<br>SEC-C_881_900<br>1.50E-11	SecA_DEAD_5_521<br>9.10E-115<br>SecA_PP_bind_353_477<br>2.40E-24<br>SecA_SW_708_929<br>1.10E-50	SecA_DEAD_8_585<br>6.50E-118<br>SecA_PP_bind_399_541<br>2.80E-35<br>SecA_SW_772_1020<br>4.10E-61<br>SEC-C_1089_1108<br>9.20E-11	SecA_DEAD
**SecB**				SecB_1_145<br>1.80E-58			
**SecM**				SecM_24_169<br>1.10E-67			
**ffh**	SRP54_N_6_82<br>3.50E-17<br>SRP54_100_295<br>2.40E-74<br>SRP_SPB_326_426<br>1.90E-32	SRP54_N_7_84<br>5.40E-17<br>SRP54_103_314<br>4.50E-65<br>SRP_SPB_345_443<br>1.40E-35	SRP54_N_7_83<br>1.20E-18<br>SRP54_101_297<br>9.40E-80<br>SRP_SPB_328_427<br>1.30E-36	SRP54_N_5_82<br>2.10E-21<br>SRP54_100_296<br>1.20E-75<br>SRP_SPB_329_427<br>1.10E-36	SRP54_N_5_82<br>2.40E-17<br>SRP54_99_295<br>1.40E-68<br>SRP_SPB_326_426<br>1.40E-26	SRP54_N_5_82<br>7.40E-16<br>SRP54_99_295<br>2.20E-76<br>SRP_SPB_326_423<br>5.20E-36	SRP_SPB
**FtsY**	SRP54_N_223_297<br>3.60E-11<br>SRP54_322_522<br>4.30E-72	SRP54_N_115_191<br>4.30E-10<br>SRP54_214_412<br>1.30E-74	SRP54_N_23_99<br>3.80E-19<br>SRP54_121_320<br>3.80E-81	SRP54_N_199_275<br>1.00E-16<br>SRP54_293_493<br>5.20E-80	SRP54_N_2_67<br>1.20E-05<br>SRP54_82_282<br>3.80E-68	SRP54_N_15_92<br>3.70E-15<br>SRP54_116_315<br>6.40E-75	SRP54_N and SRP54 (even weak signal) in absence of SRP_SPB
**SpI**	Peptidase_S24_45_126<br>3.90E-15	Peptidase_S24_100<br>179_1.10E-16	Peptidase_S24_42_110<br>1.80E-17	Peptidase_S24_86_159<br>1.70E-20<br>Peptidase_S26_260_302<br>1.40E-07	Peptidase_S24_108_194<br>1.40E-10	Peptidase_S24_31_114<br>3.00E-08	Peptidase_S24
**SpII**	Peptidase_A8_12_150<br>4.00E-41	Peptidase_A8_18_154<br>3.70E-29	Peptidase_A8_6_149<br>4.60E-44	Peptidase_A8_15_159<br>2.60E-44	Peptidase_A8_13_163<br>1.50E-30	Peptidase_A8_14_195<br>2.30E-35	Peptidase_A8

Table S1.
