# Supplementary material for: Proteins Related to the Type I Secretion System Are Associated with Secondary SecA_DEAD Domain Proteins in Some Species of Planctomycetes, Verrucomicrobia, Proteobacteria, Nitrospirae and Chlorobi
Source: PLoS One. 2015 Jun 1;10(6):e0129066. doi: 10.1371/journal.pone.0129066 (PMC4452313; doi:10.1371/journal.pone.0129066)
Supplement: S2 Table — *—protein sequence cluster; **—distance from SecA_DEAD protein; A—Alphaproteobacteria; B—Betaproteobacteria; G—Gammaproteobacteria; D/E—Delta/Epsilon subdivision of the Proteobacteria; N—Nitrospirae; P—Planctomycetes; V—Verrucomicrobia; Ph—phylum; Cl—Class. (PDF) [file pone.0129066.s016.pdf]

Cl *	D* *	GI number	Protein name*	Organism	Ph/ Cl	Locus_tag	STRING COG ID
1	1	56679293	peptidase, M50 family	Ruegeria pomeroyi DSS-3	A	SPO2714	NOG78427
1	1	117609222	peptidase, M50 family	Magnetococcus marinus MC-1	A	Mmc1_2176	NOG78427
1	1	117609244	peptidase M50	Magnetococcus marinus MC-1	A	Mmc1_2198	NOG78427
1	1	163663427	peptidase, M50 family	Methylobacterium extorquens PA1	A	Mext_2399	NOG78427
1	1	179345108	peptidase, M50 family	Methylobacterium populi BJ001	A	Mpop_2358	NOG78427
1	1	240008958	peptidase, M50 family protein	Methylobacterium extorquens AM1	A	MexAM1_META1p2401	NOG78427
1	1	82410119	peptidase, M50 family	Nitrosospira multiformis ATCC 25196	B	Nmul_A0925	NOG78427
1	1	115423020	putative membrane protein	Bordetella avium 197N	B	BAV1942	NOG78427
1	1	310762785	peptidase, M50 family	Achromobacter xylosoxidans A8	B	AXYL_04926	
1	1	158509462	peptidase, M50 family	Desulfococcus oleovorans Hxd3	D	Dole_0619	NOG78427
1	1	256578304	M50 family peptidase	Desulfomicrobium baculatum DSM 4028	D	Dbac_1342	NOG78427
1	1	333964362	secretion protein HlyD family protein	Thioalkalimicrobium cyclicum ALM1	G	Thicy_0352	
1	1	300606096	peptidase, M50 family	Candidatus Nitrospira defluvii	N	NIDE2723	
1	3	32442890	hypothetical protein-transmembrane prediction	Rhodopirellula baltica SH 1	P	RB142	NOG78427
1	4	87311840	hypothetical protein DSM3645_23885	Blastopirellula marina DSM 3645	P		
1	3	283437100	peptidase, M50 family	Pirellula staleyi DSM 6068	P	Psta_0857	
1	2	324970619	peptidase M50	Planctomyces brasiliensis DSM 5305	P	Plabr_3811	
1	2	324971532	peptidase M50	Planctomyces brasiliensis DSM 5305	P	Plabr_4739	
1	1	381386993	peptidase M50 family protein	Phycisphaera mikurensis NBRC 102666	P	PSMK_16500	
1	3	171910832	hypothetical protein VspiD_06650	Verrucomicrobium spinosum DSM 4136	V	VspiD_010100006650	
1	1	293614910	peptidase M50	Coraliomargarita akajimensis DSM 45221	V	Caka_2047	
2	5	56679297	conserved hypothetical protein	Ruegeria pomeroyi DSS-3	A	SPO2718	NOG149877
2	1	117609220	hypothetical protein Mmc1_2174	Magnetococcus marinus MC-1	A	Mmc1_2174	COG0845
2	3	117609246	secretion protein HlyD family protein	Magnetococcus marinus MC-1	A	Mmc1_2200	NOG132651
2	1	163663425	secretion protein HlyD family protein	Methylobacterium extorquens PA1	A	Mext_2397	COG0845
2	1	179345106	secretion protein HlyD family protein	Methylobacterium populi BJ001	A	Mpop_2356	COG0845
2	1	240008956	conserved hypothetical protein; putative exported protein	Methylobacterium extorquens AM1	A	MexAM1_META1p2399	COG0845
2	1	254268870	conserved hypothetical protein; putative exported protein	Methylobacterium extorquens DM4	A	METDI3179	COG0845
2	1	82410117	secretion protein HlyD	Nitrosospira multiformis ATCC 25196	B	Nmul_A0923	COG0845

2	1	115423018	putative HlyD-family secretion protein	Bordetella avium 197N	B	BAV1940	COG0845
2	1	310762783	secretion protein HlyD family protein	Achromobacter xylosoxidans A8	B	AXYL_0492 4	
2	1	158509464	secretion protein HlyD	Desulfococcus oleovorans Hxd3	D	Dole_0621	COG0845
2	1	256578306	secretion protein HlyD	Desulfomicrobium baculatum DSM 4028	D	Dbac_1344	COG0845
2	4	333964359	hypothetical protein Thicy_0349	Thioalkalimicrobium cyclicum ALM1	G	Thicy_0349	
2	3	300606092	putative Secretion protein HlyD family precursor	Candidatus Nitrospira defluvii	N	NIDE2719	
2	1	300606094	putative Secretion protein HlyD family precursor	Candidatus Nitrospira defluvii	N	NIDE2721	
2	1	32442892	probable cation efflux system	Rhodopirellula baltica SH 1	P	RB146	COG0845
2	3	87311841	probable AcrA/AcrE family protein-putative secreted protein	Blastopirellula marina DSM 3645	P		
2	1	324970622	biotin/lipoyl attachment domain-containing protein	Planctomyces brasiliensis DSM 5305	P	Plabr_3814	
2	1	324971529	biotin/lipoyl attachment domain-containing protein	Planctomyces brasiliensis DSM 5305	P	Plabr_4736	
2	1	171910828	efflux transporter, RND family, MFP subunit	Verrucomicrobium spinosum DSM 4136	V	VspiD_0101 00006630	
2	3	293614912	secretion protein HlyD family protein	Coraliomargarita akajimensis DSM 45221	V	Caka_2049	
3	2	56679294	GAF domain protein	Ruegeria pomeroyi DSS-3	A	SPO2715	NOG74050
3	2	117609223	GAF domain protein	Magnetococcus marinus MC-1	A	Mmc1_217 7	NOG74050
3	2	117609245	membrane-fusion protein-like protein	Magnetococcus marinus MC-1	A	Mmc1_219 9	COG0845
3	2	163663428	GAF domain protein	Methylobacterium extorquens PA1	A	Mext_2400	NOG74050
3	2	179345109	putative phytochrome sensor protein	Methylobacterium populi BJ001	A	Mpop_2359	NOG74050
3	2	240008959	TonB-dependent siderophore receptor	Methylobacterium extorquens AM1	A	MexAM1_M ETA1p2402	NOG74050
3	2	82410120	GAF domain protein	Nitrosospira multiformis ATCC 25196	B	Nmul_A092 6	NOG74050
3	2	115423021	putative signal transduction protein	Bordetella avium 197N	B	BAV1943	NOG74050
3	2	310762786	GAF domain protein 1	Achromobacter xylosoxidans A8	B	AXYL_0492 7	
3	2	158509461	putative phytochrome sensor protein	Desulfococcus oleovorans Hxd3	D/E	Dole_0618	NOG74050
3	2	256578303	putative phytochrome sensor protein	Desulfomicrobium baculatum DSM 4028	D/E	Dbac_1341	NOG74050
3	2	333964361	biotin/lipoyl attachment domain-containing protein	Thioalkalimicrobium cyclicum ALM1	G	Thicy_0351	
3	2	300606097	conserved protein of unknown function, contains GAF domain	Candidatus Nitrospira defluvii	N	NIDE2724	
3	2	32442891	probable membrane-fusion protein	Rhodopirellula baltica SH 1	P	RB144	COG0845
3	2	381386992	HlyD family secretion protein	Phycisphaera mikurensis NBRC 102666	P	PSMK_164 90	

3	4	171910833	probable membrane-fusion protein	Verrucomicrobium spinosum DSM 4136	V	VspiD_010100006655	
3	2	293614911	putative phytochrome sensor protein	Coraliomargarita akajimensis DSM 45221	V	Caka_2048	
4	4	56679296	SapC protein, putative	Ruegeria pomeroyi DSS-3	A	SPO2717	NOG69818
4	6	117609227	SapC family protein	Magnetococcus marinus MC-1	A	Mmc1_2181	NOG69818
4	5	117609248	SapC family protein	Magnetococcus marinus MC-1	A	Mmc1_2202	NOG69818
4	10	163663436	SapC family protein	Methylobacterium extorquens PA1	A	Mext_2408	NOG69818
4	10	179345117	SapC family protein	Methylobacterium populi BJ001	A	Mpop_2367	NOG69818
4	10	240008967	conserved hypothetical protein	Methylobacterium extorquens AM1	A	MexAM1_META1p2411	NOG69818
4	7	115423026	SapC-related protein	Bordetella avium 197N	B	BAV1948	NOG69818
4	6	310762790	SapC family protein	Achromobacter xylosoxidans A8	B	AXYL_04931	
4	6	333964357	SapC family protein	Thioalkalimicrobium cyclicum ALM1	G	Thicy_0347	
5	3	117609224	hypothetical protein Mmc1_2178	Magnetococcus marinus MC-1	A	Mmc1_2178	NOG277429
5	3	163663429	conserved hypothetical protein	Methylobacterium extorquens PA1	A	Mext_2401	NOG277429
5	3	179345110	conserved hypothetical protein	Methylobacterium populi BJ001	A	Mpop_2360	NOG277429
5	3	240008960	conserved hypothetical protein	Methylobacterium extorquens AM1	A	MexAM1_META1p2404	NOG277429
5	3	158509460	conserved hypothetical protein	Desulfococcus oleovorans Hxd3	D	Dole_0617	NOG277429
5	3	256578302	conserved hypothetical protein	Desulfomicrobium baculatum DSM 4028	D	Dbac_1340	NOG277429
5	5	87311839	hypothetical protein DSM3645_23880	Blastopirellula marina DSM 3645	P		
5	4	283437099	conserved hypothetical protein	Pirellula staleyi DSM 6068	P	Psta_0856	
