# Supplementary material for: Proteins Related to the Type I Secretion System Are Associated with Secondary SecA_DEAD Domain Proteins in Some Species of Planctomycetes, Verrucomicrobia, Proteobacteria, Nitrospirae and Chlorobi
Source: PLoS One. 2015 Jun 1;10(6):e0129066. doi: 10.1371/journal.pone.0129066 (PMC4452313; doi:10.1371/journal.pone.0129066)
Supplement: S3 Table — (PDF) [file pone.0129066.s017.pdf]

Orthologous group	Orthologous group	Gene neighbor score
COG0653	COG0845	402
COG0653	NOG78427	310
COG0653	NOG74050	286
COG0845	NOG78427	552
COG0845	NOG74050	531
NOG74050	NOG78427	853
