# Supplementary material for: Proteins Related to the Type I Secretion System Are Associated with Secondary SecA_DEAD Domain Proteins in Some Species of Planctomycetes, Verrucomicrobia, Proteobacteria, Nitrospirae and Chlorobi
Source: PLoS One. 2015 Jun 1;10(6):e0129066. doi: 10.1371/journal.pone.0129066 (PMC4452313; doi:10.1371/journal.pone.0129066)
Supplement: S4 Table — (PDF) [file pone.0129066.s018.pdf]

locus_tag ORF1	locus_tag ORF2	bOp	pOp	Distance
MexAM1_META1p2398	MexAM1_META1p2399	FALSE	0.32	70
MexAM1_META1p2399	MexAM1_META1p2400	TRUE	0.944	14
MexAM1_META1p2400	MexAM1_META1p2401	TRUE	0.925	-7
MexAM1_META1p2401	MexAM1_META1p2402	TRUE	0.961	12
MexAM1_META1p2402	MexAM1_META1p2404	FALSE	0.365	90
MexAM1_META1p1358	MexAM1_META1p1359	FALSE	0.290	124
MexAM1_META1p1359	MexAM1_META1p1360	TRUE	0.991	25
MexAM1_META1p1360	MexAM1_META1p1361	TRUE	0.958	186
MexAM1_META1p1361	MexAM1_META1p1362	TRUE	0.978	75
MexAM1_META1p1362	MexAM1_META1p1363	FALSE	0.041	269
