# Supplementary material for: Proteins Related to the Type I Secretion System Are Associated with Secondary SecA_DEAD Domain Proteins in Some Species of Planctomycetes, Verrucomicrobia, Proteobacteria, Nitrospirae and Chlorobi
Source: PLoS One. 2015 Jun 1;10(6):e0129066. doi: 10.1371/journal.pone.0129066 (PMC4452313; doi:10.1371/journal.pone.0129066)
Supplement: S5 Table — (PDF) [file pone.0129066.s019.pdf]

Gene name	Term. start	Term. end	strand	Hairpin score	Tail score	Term. sequence	Term. conf	Distance from end of gene
MexAM1_ META1p2402	2475625	2475648	-	-3.1	-3.0263	ACGATTGGCTGAACA GGGACTG-GG ACATG CCTCAACCCC TCACGGCCACCAGTG	42	26
MexAM1_ META1p2401	NONE							
MexAM1_ META1p2400	NONE							
MexAM1_ META1p2399	2470528	2470553	-	-3.2	-3.53811	TCGATGGCGGGCAAA GGTCC-ATCGC GTCGG GCGACCGGTCC CCGATCCGCCGCCTA	49	38
